# A Closed‐Loop Cardiovascular Model of the Supine‐To‐Stand Manoeuvre: Implications for Falls

**DOI:** 10.1002/cnm.70206

**Published:** 2026-08-13

**Authors:** Joeri A. van de Sande, Valeria Krzhizhanovskaya, Lex M. van Loon, Nathalie van der Velde, Eveline P. van Poelgeest, Marjolein Klop, Jurgen A. H. R. Claassen, Alfons G. Hoekstra

**Affiliations:** ^1^ Computational Science Lab, Informatics Institute, Faculty of Science University of Amsterdam Amsterdam the Netherlands; ^2^ Cardiovascular and Respiratory Physiology, TechMed Centre University of Twente Enschede the Netherlands; ^3^ Department of (Paediatric) Intensive Care University Medical Centre Utrecht Utrecht the Netherlands; ^4^ Department of Internal Medicine/Geriatrics Amsterdam University Medical Centre, University of Amsterdam Amsterdam the Netherlands; ^5^ Ageing and Later Life Amsterdam Public Health Research Institute Amsterdam the Netherlands; ^6^ Department of Geriatric Medicine Radboud University Medical Centre Nijmegen the Netherlands; ^7^ Donders Institute for Brain, Cognition, and Behaviour Radboud University Medical Centre Nijmegen the Netherlands; ^8^ Department of Cardiovascular Sciences University of Leicester Leicester UK

**Keywords:** active stand test, cardiovascular model, closed‐loop, data fitting, falls, lumped parameter, orthostatic hypotension

## Abstract

The risk of falling increases with age and orthostatic hypotension (OH) is a common cardiovascular contributor. Current OH diagnostics rely on intermittent blood pressure measurements, which may not adequately characterise haemodynamic responses during orthostatic stress. We developed a closed‐loop cardiovascular lumped‐parameter model and fitting pipeline for continuous beat‐to‐beat blood pressure and heart rate data during a supine‐to‐stand manoeuvre, enabling model‐based evaluation of orthostatic haemodynamic responses in older adults. Our 23‐compartment lumped model included baroreflex and cardiopulmonary reflex regulation and was calibrated using Finapres data from older adults (Nijmegen Radboud University PROHEALTH study). Using a stochastic ranking evolutionary strategy, we fitted 31 parameters to averaged cohort data, classifying participants as with or without a history of falls (≥ 1 fall in the previous year). The model reproduced systolic and diastolic blood pressure and heart rate responses at the population level, with normalised root mean squared errors of 10.6% and 9.6% for participants with and without a history of falls, respectively. Four model parameters differed significantly between the two population‐average fits: baroreflex reference heart rate, a baroreflex transfer function parameter, transient external muscle pressure during standing, and total blood volume. These results demonstrate that a physiologically structured cardiovascular model can reproduce population‐level orthostatic responses in older adults and highlight model‐based parameter differences associated with fall history.

## Graphical abstract

Continuous beat‐to‐beat modeling of supine‐to‐stand hemodynamics in older adults reproduces orthostatic responses and reveals differences in baroreflex, muscle‐pressure, and blood volume in those with a fall history, providing a basis for mechanistic phenotyping to enhance orthostatic hypotension diagnosis.

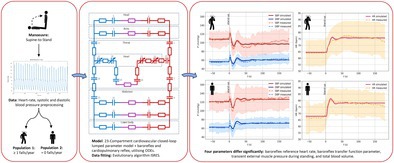

**Abbreviation**
OH: orthostatic hypotension

## 1 Introduction

Orthostatic hypotension (OH) is common in older adults and is associated with falls and syncope, as well as increased risks of all‐cause mortality, incident coronary heart disease, heart failure, and stroke [1, 2, 3, 4]. Clinically, classic OH is defined as a sustained fall in systolic blood pressure of ≥ 20 mmHg or diastolic blood pressure of ≥ 10 mmHg within 3 min of standing or during head‐up tilt [5]; variants include initial and delayed OH. Prevalence rises with age and care setting: ∼6% in middle aged adults, increasing up to ∼10%–16% in community‐dwelling adults ≥ 60 years, up to ∼50% among nursing‐home residents, and ∼70% in geriatric wards [6, 7, 8, 9, 10]. OH reflects cerebral hypoperfusion, but the underlying mechanisms are heterogeneous across individuals and are often influenced by multimorbidity and pharmacological treatment. Common contributing conditions include autonomic dysfunction (e.g., Parkinson's disease, diabetic neuropathy) and cardiovascular comorbidities such as heart failure, which can simultaneously affect multiple components of short‐term blood pressure regulation [1, 11, 12, 13, 14].

Current diagnostic practice relies on guideline‐based, event‐centred thresholds that classify OH by the magnitude of a blood‐pressure drop within predefined time windows [5]. In routine care, intermittent measurements at fixed time points are common, whereas continuous beat‐to‐beat monitoring is less frequently used. Although continuous haemodynamic recordings and variability‐based analyses are well established in autonomic and orthostatic intolerance research [15, 16] these approaches are not routinely incorporated into standard clinical assessment of OH in older adults. As a result, clinically important features of the haemodynamic response to standing, such as the rate and magnitude of the initial blood pressure drop, early compensatory mechanisms, and the recovery trajectory, may be overlooked [17, 18]. The timing of OH assessment and its association with falls remain a matter of debate [19, 20]. Active‐stand studies have highlighted the clinical relevance of early‐response windows (30–40 s), including impaired early blood‐pressure stabilisation [21, 22]. Fall‐prevention care could benefit from reducing variability in OH assessment and adopting standardised definitions of OH [23, 24]. Leveraging the full temporal profile of the response, rather than isolated points, may improve characterisation of autonomic impairment and support more personalised diagnostics.

Orthostatic hypotension reflects impaired short‐term blood‐ pressure regulation on standing. Gravitational pooling in the splanchnic and lower‐limb vasculature reduces venous return and stroke volume, while the arterial baroreflex (carotid/aortic) and low‐pressure cardiopulmonary receptors act to restore pressure via changes in heart rate, contractility, peripheral resistance, and venous tone [25, 26, 27, 28]. Comorbidities (e.g., Parkinson's disease, diabetic autonomic neuropathy, heart failure) and medications (e.g., antihypertensives, diuretics) can disrupt these reflexes, contributing to the heterogeneity of OH presentations [5, 6, 11, 14]. These mechanisms motivate our model components: hydrostatic pooling, baroreflex control, and cardiopulmonary feedback.

Several mechanistic and data‐driven modelling approaches have been proposed to study cardiovascular regulation during postural change, including models of cerebral and intracranial haemodynamics [29, 30], compartmental cardiovascular models incorporating autonomic regulation [31, 32, 33], models of heart rate regulation during postural change [34], and biomechanical simulations of the sit‐to‐stand movement [35]. Closed‐loop linear autoregressive models fitted to beat‐to‐beat cardiovascular variability have also been widely used to characterise cardiovascular control during postural stress [36, 37]. These approaches provide compact representations of baroreflex and cardiopulmonary coupling strengths during orthostatic stress, but their parameters represent system‐identification coefficients rather than explicit physiological quantities such as vascular compliance, blood volume distribution, or muscle‐pump pressure.

While these approaches collectively provide valuable mechanistic and regulatory insights, the mechanistic models are typically applied in forward‐simulation or individual‐calibration settings, and the linear parametric models characterise cardiovascular coupling within stationary epochs rather than the non‐stationary haemodynamic transition itself. Across both mechanistic and linear parametric model classes, none provide a constrained inference framework for estimating physiologically interpretable regulatory parameters at the population level, a requirement for subgroup comparison in clinically relevant cohorts such as older adults with and without a history of falls.

To overcome these limitations, the present study builds on a well‐established, full‐body baroreflex‐enabled cardiovascular modelling framework [38, 39, 40, 41, 42], which explicitly represents the systemic and pulmonary circulations, the four cardiac chambers, and baroreflex‐mediated regulatory mechanisms. Building on this foundation, we develop a model specifically tailored to the study of orthostatic blood pressure regulation in older adults and to the characteristics of continuous active‐stand measurements.

The central contribution of the present work is a constrained parameter‐inference framework applied to population‐averaged active‐stand responses, enabling the extraction and comparison of physiologically interpretable model‐based parameters reflecting cardiovascular regulatory mechanisms in older adults with and without a history of falls. Our aim is to identify dynamic model‐based parameters that differentiate between these subgroups and provide a basis for hypothesis‐generating interpretation of orthostatic haemodynamic regulation. Specifically, we (i) extend a baroreflex‐enabled lumped‐parameter model with separate cerebral and arm compartments, carotid baroreceptor sensing, transient muscle‐pressure effects, and constrained reflex actions; (ii) calibrate the model to continuous beat‐to‐beat arterial pressures and heart rate during supine‐to‐stand in older adults; and (iii) assess cohort differences in the inferred parameters.

## 2 Methods

We describe the methodology used to quantify dynamic blood‐pressure regulation during orthostatic stress. We first outline the closed‐loop cardiovascular model, then the clinical dataset and the parameter‐fitting approach (Sections 2.1 and 2.2). In accordance with Wiley's AI policy, we declare the use of artificial intelligence tools in the development for this manuscript. Specifically, we utilized Claude and ChatGPT, to assist in editing and improving the wording of the manuscript and to generate simple sections of code that were incorporated into the study. All AI‐generated content was reviewed and validated by the authors to ensure accuracy and relevance.

### 2.1 Closed‐Loop Cardiovascular Model

This subsection summarises the core mechanisms and the study‐specific extensions: (i) subdivision of the upper‐body circulation into separate cerebral and upper‐limb compartments (Section 2.1.1); (ii) inclusion of muscle‐pressure effects during active standing (Section 2.1.2); (iii) constraints (saturation/limits) on reflex control outputs (Section 2.1.3); and (iv) use of an embedded Runge–Kutta 2 (3) integrator (RK23) for time integration (Section 2.1.4).

#### 2.1.1 Model Structure

**FIGURE 1:**
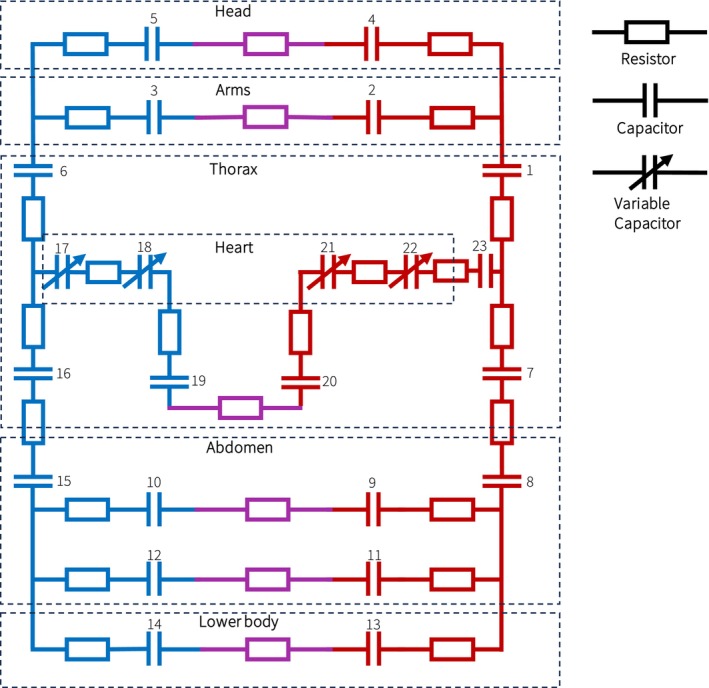
Resistor‐capacitor representation of the lumped‐parameter closed‐loop cardiovascular model. The colours represent: Red—arterial compartments, blue—venous compartments, and purple—micro vasculature. Compartments are numbered as follows: 1—upper thoracic aorta, 2—upper limb arteries, 3—upper limb veins, 4—cerebral arteries, 5—cerebral veins, 6—superior vena cava, 7—thoracic artery, 8—abdominal aorta, 9—renal arteries, 10—renal veins, 11—splanchnic arteries, 12—splanchnic veins, 13—lower body arteries, 14—lower body veins, 15—abdominal veins, 16—inferior vena cava, 17—right atrium, 18—right ventricle, 19—pulmonary arteries, 20—pulmonary veins, 21—left atrium, 22—left ventricle, and 23—ascending aorta.

The original model consisted of 21 compartments, including a single arterial and a single venous compartment for the upper body [38, 39, 41, 42]. Our data are continuous finger blood pressure recordings obtained with a Finapres device (Section 2.2.1). To reconcile this peripheral measurement with the relevant central targets, carotid‐sinus (baroreceptors) pressure and cerebral perfusion, we subdivided the upper‐body circulation into four compartments: arterial and venous for the arms, and arterial and venous for the head. This captures the hydrostatic gradients between the heart, carotid sinus, cerebral vasculature, and finger (pressure measurement). The resulting 23‐compartment structure is shown in Figure 1. We index compartments by n∈{1,…,23} as numbered in Figure 1. We include carotid baroreceptors within the arterial head compartment (n=4). Pressure to be compared with the Finapres data is obtained in the arterial upper limb compartment (n=2). In preliminary simulations, this anatomical refinement substantially improved the ability of the model to reproduce the observed cohort‐averaged responses (data not shown).

Beyond the refinement of the upper‐body circulation driven by the measurement configuration, the overall level of anatomical detail adopted in the present model reflects physiological mechanisms known to contribute to orthostatic blood pressure regulation. Redistribution of blood volume during postural change is governed by region‐specific venous compliance and vascular resistance, most prominently in the splanchnic, renal, lower‐limb and cerebral circulations. These vascular beds exhibit distinct pooling behaviour and autonomic regulation and therefore cannot be represented by a single lumped peripheral compartment without obscuring their differential contributions to arterial pressure control. The pulmonary circulation and the separation of atrial and ventricular chambers were retained to preserve realistic cardiac filling and ventricular interaction, which are required to represent preload‐dependent responses during the rapid changes in venous return following active standing. The present model therefore prioritises mechanistic interpretability of established contributors to orthostatic haemodynamics rather than minimal structural complexity.

Each compartment is characterised by a compliance (change in volume per unit pressure; C=ΔV/ΔP, or its inverse: elastance; E=1/C), an unstressed volume, and resistances for inflow and outflow. We used the classic definition of compliance [43] to calculate the pressure in a compartment based on its volume: the pressure increases with the amount of stressed volume, which is the total volume minus the unstressed volume [44]. The unstressed volume represents the baseline volume that does not contribute to transmural pressure, while the stressed volume stretches the vessel wall and generates pressure. Flow is computed by applying an analogy to Ohm's law, where the difference in pressure over a resistance determines the flow [45]. The volume is subsequently updated based on the difference in inflow and outflow (Equations (1) and (2)). The constitutive relations for the compliant compartments, solved at each time step, are as follows:

(1) $$P_{n}=\frac{V_{n}\left(t\right)-V_{n}^{u}\left(t\right)}{C_{n}\left(t\right)}+P_{r}^{\mathrm{ext}}\left(t\right),$$

(2) $$\frac{{\mathrm{dV}}_{n}\left(t\right)}{\mathrm{dt}}=q_{n}^{\text{in}}\left(t\right)-q_{n}^{\text{out}}\left(t\right)=\frac{P_{n-1}\left(t\right)-P_{n}\left(t\right)\pm P_{n}^{h}\left(t\right)}{R_{n}\left(t\right)}-\frac{P_{n}\left(t\right)-P_{n+1}\left(t\right)\pm P_{n+1}^{h}\left(t\right)}{R_{n+1}\left(t\right)},$$

where P is the transmural compartment pressure, C the compliance, V the volume, $V^{u}$ the unstressed volume, $q^{\text{in}}$ and $q^{\text{out}}$ the inflow and outflow of compartment n, and t is time. The indices n−1 and n+1 denote the compartments upstream and downstream of n, respectively. $P^{\mathrm{ext}}$ is the external pressure and $P^{h}$ is the hydrostatic pressure, which can be positive or negative depending on the direction of flow relative to gravity. The complete system of equations is given in Appendix A.

The compliances of the splanchnic, lower body and abdominal veins (n=12,14,15) are modelled using a non‐linear compliance model [46]. Venous compliance parameters reflect experimental measurements of regional venous pressure–volume relations and blood volume distributions in the lower limbs, splanchnic bed and upper body, as reported in plethysmography and morphometric studies in humans and animals [47, 48, 49, 50, 51, 52, 53]. While the model uses simplified lumped compartments, these values capture the relative distensibility and storage capacity of major venous reservoirs. Cardiac chamber volumes are derived from human measurements where available [54, 55, 56, 57] and scaled animal data for less well‐characterized chambers [58, 59]. Vascular compartment unstressed volumes are based on human measurements and literature estimates [47, 49, 52, 53, 60, 61, 62]. Unstressed volumes and inflow resistances of the upper limb, cerebral, renal, splanchnic, and lower body veins (n=3, 5, 10, 12, 14) are modulated by the baroreflex and cardiopulmonary reflex (Section 2.1.3).

We adapt the model originally developed for healthy younger individuals [39, 42] by adjusting the peripheral resistances (compartments n=3, 5, 10, 12, 14) and all compliances, except those of the heart, to reflect the physiological characteristics of older adults. Based on literature values, new baseline values (prior to data fitting) are computed by applying age‐related changes. Specifically, vascular elastance and peripheral resistance are adjusted using average annual increases of 1.58% and 1.11%, respectively [63]. Assuming an average age difference of 30 years, we apply age‐based scaling factors for elastance of $1.0158^{30}\approx 1.6$ and for peripheral resistance of $1.0111^{30}\approx 1.4$. We note that the applied age‐based adjustments to vascular elastance and resistance are deterministic and represent average population‐level changes. In reality, ageing‐related vascular remodelling is heterogeneous across individuals and vascular beds, which our model does not capture. We consider this approach appropriate for establishing initial baseline values and parameter bounds for older adults in a population‐average sense, while subsequent optimization to experimental data allows individualized parameter tuning within physiologically plausible ranges. Capturing bed‐specific heterogeneity would introduce additional parameters that are unlikely to be identifiable from the available data.

The cardiac chambers are modelled using a time‐varying elastance formulation, in which the atria and ventricles are represented as compliant compartments with periodically varying elastance [39]. Separate activation functions are defined for the atria (n={17,21}; right and left atrium) and the ventricles (n={18,22}; right and left ventricle). Smooth transitions between minimum and maximum elastance are generated using sinusoidal functions, consistent with classical formulations of cardiac activation [64]. A temporal offset between atrial and ventricular activation is introduced to reproduce the physiological delay between atrial and ventricular contraction [65].

**FIGURE 2:**
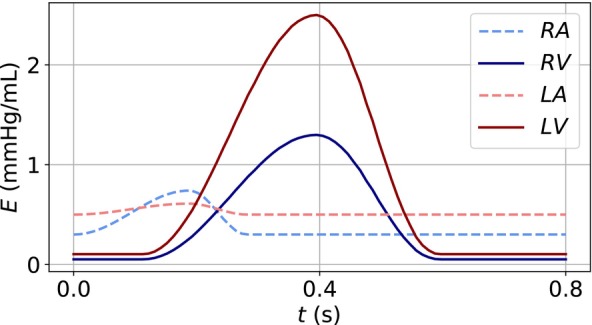
Plot of cardiac elastance (E) vs. time (t) for one cardiac cycle in the four heart chambers: RA—right atrium, RV—right ventricle, LA—left atrium, LV—left ventricle.

Cardiac valves are represented as pressure‐gated unidirectional flow elements between the cardiac chambers and the connected vascular compartments, such that backflow is prohibited and isovolumetric contraction and relaxation phases emerge naturally. The duration and timing of the atrial and ventricular activation phases are dynamically adapted to the cardiac cycle length. Specifically, all characteristic timing parameters are scaled with the square root of the heart period (HP), following Bazett's formulation [66], such that

$$T_{X}=k_{X}\sqrt{\mathrm{HP}},$$

where $k_{X}$ denotes a compartment‐specific scaling constant. For each atrial compartment, the time‐varying elastance is defined as

(3) $$E_{n}\left(t\right)=\left\{\begin{array}{ll} \frac{1}{2}\Delta E_{n}\left(1-\cos\left(\frac{\pi t}{T_{\mathrm{AV}}}\right)\right)+E_{n}^{\min}, & 0\leq t\leq T_{\mathrm{AV}},\\ \frac{1}{2}\Delta E_{n}\left(1+\cos\left(\frac{2\pi t}{T_{\mathrm{AV}}}\right)\right)+E_{n}^{\min}, & T_{\mathrm{AV}}<t\leq \frac{3}{2}T_{\mathrm{AV}},\\ E_{n}^{\min}, & \frac{3}{2}T_{\mathrm{AV}}<t\leq T, \end{array}\right.$$

and for each ventricular compartment as

(4) $$E_{n}\left(t\right)=\left\{\begin{array}{ll} \frac{1}{2}\Delta E_{n}\left(1-\cos\left(\frac{\pi \left(t-T_{\mathrm{SA}}\right)}{T_{\mathrm{SV}}}\right)\right)+E_{n}^{\min}, & T_{\mathrm{SA}}\leq t\leq T_{S},\\ \frac{1}{2}\Delta E_{n}\left(1+\cos\left(\frac{2\pi \left(t-T_{S}\right)}{T_{S}}\right)\right)+E_{n}^{\min}, & T_{S}<t\leq \frac{3}{2}T_{S},\\ E_{n}^{\min}, & 0\leq t<T_{\mathrm{SA}}\;\text{or}\;\frac{3}{2}T_{S}<t\leq T. \end{array}\right.$$

here $\Delta E_{n}=E_{n}^{\max}-E_{n}^{\min}$, with $E_{n}^{\max}$ and $E_{n}^{\min}$ denoting the maximal and minimal elastance of compartment n, respectively. The cardiac cycle length is denoted by T. For the ventricles, $T_{S}=T_{\mathrm{SV}}+T_{\mathrm{SA}}$, where $T_{\mathrm{SA}}$ represents the atrioventricular delay and $T_{\mathrm{SV}}$ the duration of ventricular activation. A schematic illustration of the resulting atrial and ventricular elastance waveforms is shown in Figure 2. While more detailed atrial activation models with more complex temporal dynamics have been proposed (see e.g., [67, 68]), we adopt a simplified atrial elastance formulation with a single characteristic atrial activation time to limit parameter dimensionality given the available data.

#### 2.1.2 Supine‐To‐Stand Manoeuvre

**FIGURE 3:**
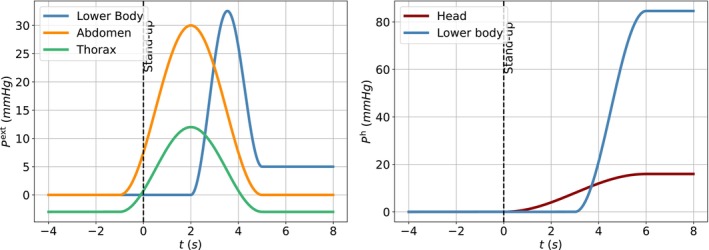
Time series of the exerted external muscle pressure $P_{\mathrm{ext}}$ (left, Equation (7)) and hydrostatic pressure magnitude $P^{h}$ (right, Equation (6)) during the stand‐up manoeuvre. In the external muscle pressure graph, the lower body, abdominal and thoracic regions correspond to the compartment sets ${ℛ}_{\mathrm{lb}}=\left\{13,14\right\}$, ${ℛ}_{\mathrm{abd}}=\left\{8,9,10,11,12,15\right\}$ and ${ℛ}_{\text{thrx}}=\left\{1,6,7,16,17,18,19,20,21,22,23\right\}$, respectively. Although all compartments are subjected to hydrostatic pressure, only representative traces are shown to illustrate the different onset timing in the lower‐body compartments and the different amplitudes arising from the effective vertical lengths of the head and leg compartments, ${ℛ}_{\text{head}}=\left\{4,5\right\}$ and ${ℛ}_{\mathrm{lb}}=\left\{13,14\right\}$. Although a positive $P^{h}$ is shown for both head and lower‐body compartments, due the direction of the flow, the resulting effect is a reduction of effective perfusion pressure towards the head and an increase towards the lower extremities after standing.

The postural change performed by a subject is modelled by introducing hydrostatic ($P_{n}^{h}$) and external ($P_{r}^{\mathrm{ext}}$) pressures into the model (Equations (1) and (2)), as shown in Figure 3. The applied external pressures emulate the effect of skeletal muscle contraction on venous return, that is, the muscle pump [52, 69]. While absolute pressures are initially estimated from younger adult studies and adapted via parameter fitting, this approach captures the general physiological effect of muscle‐induced augmentation of venous return during standing. The function $\alpha_{m}\left(t\right)$ with m∈{1,2} is used to define permanent ($\alpha_{1}\left(t\right)$) and temporary ($\alpha_{2}\left(t\right)$) activation:

(5) $$\alpha_{m}\left(t\right)=\left\{\begin{array}{ll} 0 & \text{if}\;t<t_{0},\\ \frac{1}{2}\left(1-\cos\left(m\cdot \pi \cdot \frac{t-t_{0}}{T_{\text{stand}}-t_{0}}\right)\right) & \text{if}\;t_{0}\leq t\leq T_{\text{stand}},\\ \alpha_{m}\left(T_{\text{stand}}\right) & \text{if}\;t>T_{\text{stand}}, \end{array}\;\text{with}\;m\in \left\{1,2\right\}\right.$$

where $t_{0}$ is the start time of the standing manoeuvre and $T_{\text{stand}}$ the duration of the transition from supine to standing. In our case, we fix the transition duration at 6 s. Average and median standing times in older adult populations have been reported ranging from 5 to 7 s [70, 71, 72], while population studies generally ask participants to stand up quickly, within 5 s [24]. Individual stand‐up durations are available and will be used in subject‐specific model fitting.

From Equation (5), the compartmental hydrostatic pressure ($P_{n}^{h}$, Bernoulli) and the external pressure ($P_{r}^{\mathrm{ext}}$) are calculated as:

(6) $$P_{n}^{h}\left(t\right)=\rho \cdot g\cdot h_{n}\cdot \sin\left(\frac{\pi }{2}\cdot \alpha_{1}^{h}\left(t\right)\right),$$

(7) $$P_{r}^{\mathrm{ext}}\left(t\right)=P_{r}^{\mathrm{ext},0}+P_{r}^{\mathrm{ext},\text{perm}}\cdot \alpha_{1,r}\left(t\right)+P_{r}^{\mathrm{ext},\text{temp}}\cdot \alpha_{2,r}\left(t\right),$$

where ρ is the density of blood, g the gravitational acceleration, $h_{n}$ the vertical distance of compartment n from the previous compartment. $P_{r}^{\mathrm{ext},\text{perm}}$ and $P_{r}^{\mathrm{ext},\text{temp}}$ in Equation (7) are the amplitudes of the permanent and momentary components of the external pressure, respectively. $P_{r}^{\mathrm{ext},0}$ is the ever‐present external pressure, regardless of position. Total external pressure ($P_{r}^{\mathrm{ext}}$) is applied to the compartments of the thorax, abdomen, and lower body (see Figure 1). We define the thorax (thrx), abdomen (abd) and lower body (lb) regions (r) as ${ℛ}_{\text{thrx}}=\left\{1, 6, 7, 16, 17, 18, 19, 20, 21, 22, 23\right\},\;{ℛ}_{\mathrm{abd}}=\left\{8, 9, 10, 11, 12, 15\right\},\;{ℛ}_{\mathrm{lb}}=\left\{13,14\right\}.$ The magnitude and timing of the pressure components differ per group. The parameter values are given in Table 1. The hydrostatic term $P^{h}$ represents the magnitude of the gravitational pressure contribution for each compartment. Its effect on the governing equations is direction dependent: depending on the orientation of the flow path, the hydrostatic contribution is added to or subtracted from the local pressure drop.

**TABLE 1:** Region‐specific timing and external pressure parameters of Equations (5) and (7).

Region	$\Delta t_{0}$ (s)	$P_{r}^{\mathrm{ext},0}$ (mmHg)	$P_{r}^{\mathrm{ext},\text{perm}}$ (mmHg)	$P_{r}^{\mathrm{ext},\text{temp}}$ (mmHg)
Thorax	−1	−3.0 [73]	0.0	15.0 [74]
Abdomen	−1	0.0	0.0	30.0 [75]
Lower body	$+T_{\text{stand}}/2$	0.0	5.0	30.0

*Note:* $\Delta t_{0}$ is the onset difference compared to the start of stand‐up $t_{0}$, $P_{r}^{\mathrm{ext},0}$ is the ever‐present pressure, $P_{r}^{\mathrm{ext},\text{perm}}$ is the permanent pressure due to stand‐up, $P_{r}^{\mathrm{ext},\text{temp}}$ is the temporary pressure due to stand‐up, $T_{\text{stand}}$ is the duration of the supine‐to‐stand transition.

The two‐phase division of the supine‐to‐stand manoeuvre is intended to reflect the known biomechanics of active standing. During the initial phase, the thorax and abdomen are engaged to stabilise posture and initiate leg extension, while the lower body remains relatively passive. In the second phase, the lower‐limb muscles are actively recruited to complete the standing movement, producing transient increases in intra‐abdominal and lower‐body pressure. These timing differences in muscle engagement and resulting pressure changes are consistent with observations in sit‐to‐stand studies in young adults, which demonstrate sequential activation of upper‐ and lower‐body musculature [35, 76]. Inter‐individual variability in the biomechanics of standing is observed [35]. While direct measurements in older adults are scarce, we use these biomechanical principles to guide the temporal partitioning of external pressure, and initial magnitudes are subsequently adapted via parameter fitting to the observed haemodynamic responses. This approach allows the model to capture the differential onset and amplitude of external pressures across body regions, providing a physiologically motivated, rather than purely arbitrary, representation of muscle‐induced pressure changes during standing.

Because we observed an increase in blood pressure already before the start of stand‐up, we assume that muscle strain occurs 1 s before the actual stand‐up onset. And while test subjects changed their position from supine to sitting before engaging their legs to fully stand, we divide the manoeuvre into two phases. The second phase, beginning at $t_{0}+T_{\text{stand}}/2$ s, accounts for external pressure and rotation of the lower body, where $T_{\text{stand}}=6s$ (Equation (5)).

$P_{\mathrm{abd}}^{\mathrm{ext},\text{temp}}$ is based on findings by Tanaka et al. who reported a sharp increase in intra‐abdominal pressure (43 ± 22 mmHg) upon standing, caused by contraction of the leg and abdominal muscles in seven individuals aged 24–41 years [75]. We selected a maximal temporary pressure of 30 mmHg as the initial guess for data fitting, based on the expectation that older adults generally have less muscle mass. In physiological studies without specific breathing instructions, an immediate increase in blood pressure was observed, ranging from 10 to 28 mmHg for systolic and around 10 mmHg for diastolic pressure [74, 77, 78]. For the thorax, we used 15 mmHg as the initial guess for the maximal temporary pressure, as this corresponds to the pressure increase observed in the population‐averaged systolic and diastolic blood pressures and is at the lower end of the range reported in these studies. We use Daly and Bondurant's estimate (of −3.0 ± 0.5 mmHg) for intrathoracic pressure [73]. Finally, we assume the temporary pressure in the lower body to be the same as in the abdomen, and estimate the permanently exerted standing muscle pressure to be 5 mmHg. These values are used as the initial guesses for the corresponding parameters.

#### 2.1.3 Reflexes

The reflex dynamics are represented using effector‐specific impulse response kernels, following the regulatory set‐point formulation introduced by Heldt et al. [39]. Rather than describing the reflex pathways by additional state‐space differential equations, the present model computes effector responses by convolving a finite discrete history of the effective pressure error with physiologically motivated kernels. This formulation can be interpreted as the input–output representation of an underlying linear time‐invariant dynamical system driven by the pressure error signal. The kernel‐based approach explicitly represents the distributed delay and temporal dispersion arising from afferent transmission, central processing, efferent neural conduction, and effector organ dynamics. In contrast to a single fixed delay, the reflex latency is embedded in the shape and support of the kernels. Separate kernels are used for parasympathetic and sympathetic pathways to reflect their markedly different time courses: the parasympathetic response is rapid (latency ∼0.5–1 s), whereas the sympathetic response exhibits a slower onset (∼2 s) and a prolonged action over several tens of seconds. This formulation allows the temporal characteristics of the reflex, such as latency, rise time, and decay, to be specified directly from experimental observations without introducing additional state variables that are difficult to identify from the available data.

The baroreflex and cardiopulmonary response modelling pathway starts with receptors sensing a pressure difference from a reference pressure set‐point $P_{s}^{\text{setp}}$. This signal is then scaled to obtain the resulting scaled pressure difference [38]:

(8) $$\delta {\bar{P}}_{s}\left[i\right]=P_{s}^{\mathrm{sc}}\cdot \tan^{-1}\left(\frac{{\bar{P}}_{s}\left[i\right]-P_{s}^{\text{setp}}}{P_{s}^{\mathrm{sc}}}\right),$$

where $P_{s}^{\mathrm{sc}}$ is a shape parameter that controls the saturation rate of the pressure sensors (see Figure 6). Here, s∈ {car, ra} indexes the sensing site (arterial/carotid vs. right atrial/central venous). The term ${\bar{P}}_{s}\left[i\right]$ represents a moving average of the transmural pressure over the most recent 0.25 s, which smooths the fast pulsations of the pressure waveform. This moving average is updated every 1/16 s (16 Hz), so that consecutive averages overlap. The index i therefore denotes the discrete reflex update step of this filtered signal (updated at 16 Hz), and should be distinguished from the continuous‐time index of the ODE integration. The inverse tangent accounts for the experimentally observed sigmoid reflex behaviour that saturates the response [79].

A temporal weighting, including an effective delay, is applied to the scaled pressure difference from Equation (8) [39]. Reflex responses are computed by convolving a finite discrete history of the effective pressure error with normalised impulse response kernels $T_{r}$. These kernels determine the time window over which past pressure deviations contribute to the reflex response. The reflex input is therefore given by

(9) $$\Delta X_{r}\left[i\right]=\sum_{k=0}^{N_{r}-1}T_{r}\left[k\right]\;\delta {\bar{P}}_{s\left(r\right)}\left[i-k\right],$$

where i is the discrete time index, r indexes the reflex pathway, s(r) indicates the mapping from reflex to sensing site, and $N_{r}$ denotes the kernel length. The pressure history $\delta {\bar{P}}_{s\left(r\right)}$ is stored in a finite buffer representing the recent time history of the sensed pressure signal of 60 s sampled at 0.0625 s intervals, resulting in a discrete signal of 960 elements. Each kernel $T_{r}\left[k\right]$ is constructed as a normalised triangular impulse response function. The kernel increases linearly from zero at $i_{\text{start}}$ to a maximum at $i_{\text{peak}}$ and subsequently decreases linearly to zero at $i_{\mathrm{end}}$:

(10) $$T_{r}\left[k\right]=\left\{\begin{array}{ll} \frac{k-i_{\text{start}}}{i_{\text{peak}}-i_{\text{start}}}, & i_{\text{start}}\leq k<i_{\text{peak}}\\ \frac{i_{\mathrm{end}}-k}{i_{\mathrm{end}}-i_{\text{peak}}}, & i_{\text{peak}}\leq k<i_{\mathrm{end}}\\ 0, & \text{otherwise}. \end{array}\right.$$

**FIGURE 4:**
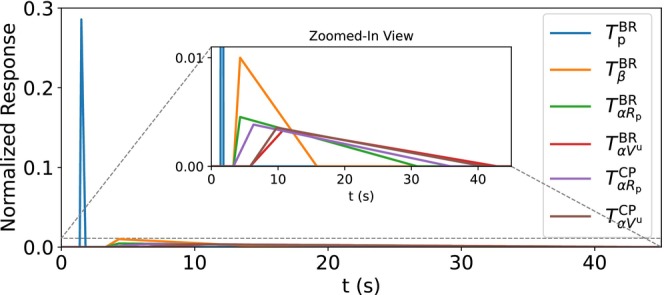
Normalised convolution kernels [39] used to specify the period of the baroreflex (BR) and cardiopulmonary (CPR) reflex effect. Traces correspond to different autonomic efferent pathways: Parasympathetic (*p*), β‐adrenergic (β), α‐adrenergic acting on arteriolar resistance ($\alpha R_{p}$), and on venous unstressed volume ($\alpha V^{u}$). (Equation (9)).

The kernel is subsequently normalised such that

(11) $$\sum_{k=0}^{N_{r}-1}T_{r}\left[k\right]=1,$$

ensuring that the convolution scales the pressure deviation without altering its overall magnitude. The integers $i_{\text{start}}$, $i_{\text{peak}}$, and $i_{\mathrm{end}}$ define the onset, maximum, and termination of the triangular kernel, respectively (Figure 4; Appendix F Table A8). The triangular shape provides a simple phenomenological representation of the temporal dynamics of autonomic reflex responses, capturing a finite response latency (onset), a gradual recruitment toward a peak effect, and a subsequent decay as the stimulus is removed. This form approximates experimentally observed baroreflex impulse responses, which exhibit delayed, transient dynamics over a limited time window, while remaining simple and identifiable from the available data. The model includes parasympathetic, β‐sympathetic, and α‐sympathetic reflex pathways originating from either the carotid sinus or right atrium and acting on cardiac and vascular effectors. The implemented sensing–effector pathways are summarised in Table 2. The kernel timing parameters were adopted from human carotid‐sinus and neck‐pressure studies for parasympathetic and vascular sympathetic responses, and from canine experiments for β‐sympathetic and venous tone dynamics [80, 81, 82, 83, 84, 85, 86, 87, 88, 89].

**TABLE 2:** Summary of implemented reflex pathways, sensing sites and effectors.

Reflex pathway	Sensing site	Effector	Controlled variable
Parasympathetic (BR)	Carotid sinus pressure	Sinoatrial node	Heart period (HP)
β‐sympathetic (BR)	Carotid sinus pressure	Ventricular myocardium (LV, RV)	Minimum ventricular compliance $C^{\min}$
β‐sympathetic (BR)	Carotid sinus pressure	Sinoatrial node	Heart period (HP)
α‐sympathetic (BR)	Carotid sinus pressure	Peripheral arterioles (regional)	Vascular resistance $R_{e}$
α‐sympathetic (BR)	Carotid sinus pressure	Peripheral veins (regional)	Unstressed volume $V_{e}^{u}$
α‐sympathetic (CPR)	Right atrial pressure	Peripheral arterioles (regional)	Vascular resistance $R_{e}$
α‐sympathetic (CPR)	Right atrial pressure	Peripheral veins (regional)	Unstressed volume $V_{e}^{u}$

Abbreviations: BR, Arterial baroreflex (carotid sinus); CPR, Cardiopulmonary reflex (right atrium).

The kernel‐weighted contributions $\Delta X_{r}\left[i\right]$ are multiplied by their corresponding gains and added to a setpoint value [39]. In general form, the regulated effector Y can be written as

(12) $$Y_{e}\left[i\right]=Y_{e}^{\text{setp}}+\sum_{r}G_{e,r}\;\Delta X_{r}\left[i\right],$$

where $Y_{e}\left[i\right]$ denotes the regulated effector variable, which may represent heart period (HP), minimum ventricular compliance ($C^{\min}$), vascular resistance ($R_{e}$), or unstressed volume ($V_{e}^{u}$). The index e identifies the effector and, where applicable, the compartment. For ventricular compliance $C_{e}$, e∈{18,22} corresponds to the right and left ventricles (RV, LV), respectively. For vascular resistance $R_{e}$ and venous unstressed volume $V_{e}^{u}$, e∈{3, 5, 10, 12, 14} corresponds to the upper‐limb, cerebral, renal, splanchnic, and lower‐body vascular compartments. The gains $G_{e,r}$ are reflex‐specific, representing baroreflex (BR) or cardiopulmonary reflex (CPR) pathways, and are non‐zero only for reflex–effector combinations that are physiologically active. Expanding Equation (12), the combined baroreflex and cardiopulmonary reflex contributions to each effector are

(13) $$\mathrm{HP}\left[i\right]={\mathrm{HP}}^{\text{setp}}+\Delta X_{p}^{\mathrm{BR}}\left[i\right]\;G_{\mathrm{HR}}^{\text{paras}}+\Delta X_{\beta }^{\mathrm{BR}}\left[i\right]\;G_{\mathrm{HR}}^{\text{symp}},$$

(14) $$C_{\mathrm{LV}}^{\min}\left[i\right]=C_{\mathrm{LV}}^{\text{setp}}+\Delta X_{\beta }^{\mathrm{BR}}\left[i\right]\;G_{C_{\mathrm{LV}}}^{\mathrm{BR}},$$

(15) $$C_{\mathrm{RV}}^{\min}\left[i\right]=C_{\mathrm{RV}}^{\text{setp}}+\Delta X_{\beta }^{\mathrm{BR}}\left[i\right]\;G_{C_{\mathrm{RV}}}^{\mathrm{BR}},$$

(16) $$R_{e}\left[i\right]=R_{e}^{\text{setp}}+\Delta X_{\alpha R_{p}}^{\mathrm{BR}}\left[i\right]\;G_{R_{p}}^{\mathrm{BR}}+\Delta X_{\alpha R_{p}}^{\mathrm{CPR}}\left[i\right]\;G_{R_{p}}^{\mathrm{CPR}},$$

(17) $$V_{e}^{u}\left[i\right]=V_{e}^{u,\text{setp}}+\Delta X_{\alpha V^{u}}^{\mathrm{BR}}\left[i\right]\;G_{V^{u},e}^{\mathrm{BR}}+\Delta X_{\alpha V^{u}}^{\mathrm{CPR}}\left[i\right]\;G_{V^{u},e}^{\mathrm{CPR}}.$$

The static gains of the arterial baroreflex are assigned following experimental and modelling studies [39]. Heart period gains are derived from R–R interval responses to step changes in arterial pressure [90, 91, 92], pooled as in Heldt [39] and split equally between sympathetic and parasympathetic pathways [38]. Left and right ventricular contractility gains follow the reasoning of Davis [93]. Peripheral resistance gains reflect the capacity of total peripheral resistance to increase up to 2.5–3.0 PRU under orthostatic stress [94, 95], and changes in venous unstressed volume are informed by dog experiments showing a maximum systemic reservoir change of 12 mL/kg, primarily from the abdominal compartment [96]. The arterial baroreflex set‐point pressure is set to 81 mmHg, 10 mmHg lower than Heldt [39], reflecting the shift of the receptors from the aorta to the carotid sinus.

CPR gains follow Heldt et al. [39]. The CPR‐induced change in heart period is set to zero, reflecting the negligible coupling of right atrial pressure to heart rate, consistent with the Bainbridge reflex [97, 98, 99]. CPR effects on peripheral resistance are based on lower‐body negative pressure experiments [100], which quantify the baroreflex‐independent vascular responses to central blood volume shifts. Venous unstressed volume changes are informed by human haemorrhage/splanchnic studies [101]. The right atrial set‐point is 5 mmHg [39], and CPR kernel timing matches that of the arterial baroreflex [39].

We constrain venous unstressed volumes and peripheral arterial resistances regulated by the baroreflex and cardiopulmonary reflex to ±3 orders of magnitude (×1000) about their nominal values. These deliberately broad bounds avoid constraining solutions within physiological ranges, while improving numerical stability by preventing the optimiser from exploring non‐physiological parameter sets that can drive peripheral resistance towards zero or cause oscillatory divergence in reflex control.

#### 2.1.4 Initialisation and Numerical Integration

The model is initialised using physiological parameter values (Appendix F Tables A4, A5, A6, A7, A8, A9, A10). For a selection of these parameters, these values serve as initial parameter estimates and are subsequently refined through parameter estimation using experimental data (Section 2.2).

Initial compartment volumes are obtained by distributing the total blood volume $V_{\mathrm{tot}}$ across compartments $V_{n}$ according to their relative unstressed volumes $V_{n}^{u}$:

(18) $$V_{n}=V_{\mathrm{tot}}\cdot \frac{V_{n}^{u}}{\sum_{i}V_{i}^{u}}.$$

These initial values determine the starting pressures of the model compartments. Subsequent dynamics are governed primarily by cardiac pumping, baroreflex regulation, and vascular resistances (Equations (1) and (2)).

We integrate the system of ordinary differential equations using the adaptive RK23 method [102]. At each step, the solver enforces $\text{error}\leq {\mathrm{tol}}_{\mathrm{abs}}+{\mathrm{tol}}_{\mathrm{rel}}\cdot |y|$. Here, |y| denotes the magnitude of the vector of model state variables used for adaptive error scaling. During integration, relative and absolute tolerances are set to ${\mathrm{tol}}_{\mathrm{abs}}={\mathrm{tol}}_{\mathrm{rel}}=10^{-4}$. Because the reflex functions can influence parameters for up to 60 s, an additional 120 s of simulation is performed to allow the reflex dynamics to stabilise. The supine‐to‐stand manoeuvre is introduced by applying time‐dependent hydrostatic and external pressures to the relevant compartments (Section 2.1.2). The total simulated period, including initialisation, rest, manoeuvre, and standing, is 360 s. On a single CPU core (no GPU acceleration or parallelization), a single simulation requires approximately 200 s of wall‐clock time.

### 2.2 Model Fitting to Clinical Data

We fitted the enhanced 23‐compartment, closed‐loop cardiovascular model to cohort‐average systolic and diastolic blood pressure and heart rate from older adults performing a supine‐to‐stand manoeuvre. Below, we describe the clinical dataset, pre‐processing, objective function and weighting, the optimisation algorithm and settings, parameter tying, and the multi‐start strategy used to assess robustness.

#### 2.2.1 Clinical Data

We use the PROHEALTH dataset [103, 104], which provides time‐resolved blood‐pressure measurements recorded using a photoplethysmographic monitor (Finapres Medical Systems BV, The Netherlands) in older individuals performing standardised postural changes. Ethical approval was obtained from the Medical Ethics Committee (CMO Arnhem–Nijmegen). All participants provided written informed consent. The study was performed in accordance with the Declaration of Helsinki. The present study uses fully de‐identified data for model calibration.

From these beat‐to‐beat measurements, systolic and diastolic blood pressure and heart rate were derived. The dataset includes 30 participants aged 65 years and older (mean age 74±7 years), of whom 11 (37%) were women. Falls were defined as “an unexpected event in which an individual comes to rest on the ground, floor, or a lower level”. Fall history was self‐reported at baseline and considered positive if ≥ 1 fall occurred in the previous year [103, 104]. Seven participants met this criterion; 23 did not.

**FIGURE A1:**
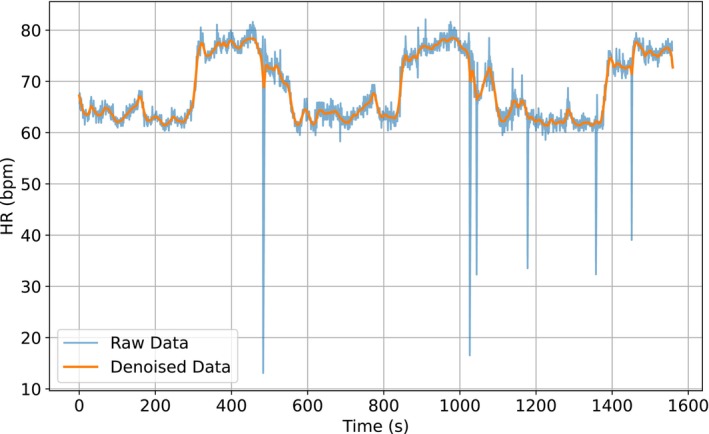
Comparison of pressure derived heart rate (raw) and cleaned heart rate (HR) time series recorded from one individual performing three stand‐up manoeuvres at *t* = 300 (s), 850 (s), 1350 (s), respectively.

For the present analysis, we considered only the first supine‐to‐stand manoeuvre from each participant. Heart rate was derived by calculating the time interval between successive systolic blood‐pressure peaks (peak‐to‐peak method). Pre‐processing comprised: heart rate outlier detection in using an Isolation Forest (contamination =0.08) and removal; interpolation of missing values; and application of a first‐order Butterworth filter (cutoff =0.05 Hz) to remove high‐frequency noise (Appendix B, Figure A1). Time traces were aligned to the stand‐up event and cropped to 60 s before and 180 s after standing. Population averages and standard deviations were then calculated separately for participants with and without a history of falls. The use of population‐averaged responses is intended as an initial step to establish the behaviour of the modelling framework and to assess whether robust population‐level differences are detectable, rather than to replace subject‐specific model calibration. Because the model is non‐linear, fitting to population‐averaged responses does not yield averages of subject‐specific physiological parameters. The estimated parameters should therefore be interpreted as cohort‐level regulatory phenotypes that reproduce the characteristic haemodynamic response of each cohort. Population averaging is used here to reduce measurement noise and timing variability during the stand manoeuvre and to enable a stable and controlled comparison between participants with and without a history of falls under an identical model and inference framework.

#### 2.2.2 Objective Function and Normalisation

To reduce variable‐scale bias, we applied min–max normalisation per variable (systolic and diastolic blood pressure and heart rate), which preserves the full range of observed dynamics without assuming a specific distribution. For a given variable, let $a_{\text{data}}$ denote the data time series and $a_{\text{model}}$ the corresponding model output. Define

$$a_{\min}=\min\left(a_{\text{data}}\right),\;a_{\max}=\max\left(a_{\text{data}}\right),$$

and the normalised series

$$n_{\text{data}}=\frac{a_{\text{data}}-a_{\min}}{a_{\max}-a_{\min}},\;n_{\text{model}}=\frac{a_{\text{model}}-a_{\min}}{a_{\max}-a_{\min}}.$$

The root‐mean‐square error (RMSE) between normalised data and model was computed per variable. A weighting factor of two was applied to the first 60 s after stand‐up (the interval during which the largest changes occur), and the first 120 s of each simulation (reflex stabilisation) were excluded from the objective calculation.

#### 2.2.3 Optimiser and Settings

We used the Improved Stochastic Ranking Evolutionary Strategy (ISRES) [105, 106] to minimise the weighted RMSE subject to bound and inequality constraints, chosen for robustness on constrained, non‐convex, derivative‐free problems [107]. We used Pymoo [108] with selection ratio 1/7, mutation γ=0.7, recombination α=0.6, and 150 generations of 288 offspring; termination occurred at the generation limit. Settings were chosen based on observed convergence (details shown in Appendix D, example convergence in Figure A6, all final generation RMSE shown in Table A1).

To balance speed and accuracy, we used a two‐stage solver‐tolerance schedule: ${\mathrm{tol}}_{\mathrm{abs}}={\mathrm{tol}}_{\mathrm{rel}}=10^{-3}$ for the first 20 generations, then $10^{-4}$ thereafter (see Section 2.1.4 for integrator details), yielding an approximately 2.5× speed‐up during the early optimisation phase.

#### 2.2.4 Model Parameters

The closed‐loop cardiovascular model (Section 2.1) contains a large number of physiological parameters describing vascular properties, cardiac mechanics, and reflex dynamics. To obtain a tractable optimisation problem and avoid over‐parametrisation relative to the available data, several parameters were grouped and estimated jointly. Peripheral resistances in the head, arms, liver, kidneys, and lower body were optimised via a single scaling parameter; all resistances, all compartmental unstressed volumes, all arterial compliances and all venous compliances were scaled by a global multiplier to retain relative vascular heterogeneity. Similarly, previously defined compartment‐specific arterial and venous reflex gains, modulating the unstressed volumes, were modulated by global scaling parameters, such that their relative values across vascular territories remained fixed.

Parameter selection was further guided by a previously published local sensitivity analysis of the same cardiovascular model [109], in which 107 parameters were evaluated with respect to their influence on mean arterial pressure, central venous pressure, stroke volume, and heart rate. That analysis demonstrated that approximately 70%–80% of parameters exert negligible influence on model outputs, whereas a small subset of parameters dominates system behaviour. The most sensitive parameters were associated with blood volume regulation, baroreflex and cardiopulmonary reflex set‐points, heart rate control, ventricular compliance, and ventricular systolic timing. The parameters selected for optimisation in the present study were therefore chosen to represent these dominant physiological mechanisms, including reflex gains and set‐points, ventricular elastance parameters, vascular resistances, and blood volume scaling terms. Ventricular systolic timing parameters were not included in the optimisation and were kept at literature values, as variations in these parameters can introduce non‐smooth changes in the time‐varying elastance function and are difficult to identify reliably from the available haemodynamic observations.

This parameter grouping results in a total of 31 estimated parameters (Appendix E, Table A3), while the remaining physiological parameters were fixed to literature‐based values. A detailed list of fitted parameters and their ranges is provided in Table A3, with baseline physiological values listed in Appendix F Tables A4, A5, A6, A7, A8, A9, A10.

Given the number of observed outputs (systolic and diastolic blood pressure and heart rate during a stand‐up) relative to the number of fitted parameters, and the closed‐loop non‐linear structure with delays and saturations, the present model is not expected to be fully structurally or practically identifiable, consistent with previous studies on lumped‐parameter cardiovascular models and resistor–capacitor networks [110, 111]. The estimated parameters should therefore be interpreted as model‐based indicators of regulatory mechanisms that reproduce the observed cohort‐level haemodynamic responses, rather than as uniquely identifiable physiological quantities. Finally, the analysis is not intended to infer region‐specific autonomic dysfunction, but rather to assess whether system‐level regulatory parameters exhibit consistent group differences under identical model assumptions.

#### 2.2.5 Multi‐Start and Robustness

For each population (with and without a history of falls), we performed 10 independent optimisation runs with different random seeds. In the stochastic ISRES algorithm, the seed determines both the initial population and the subsequent evolutionary search trajectory, including mutation, recombination, and selection processes. Because the objective landscape is multimodal, runs may converge to different local optima; multiple seeds therefore allow us to assess robustness with respect to stochastic initialisation and search dynamics. Ten seeds were found to be sufficient to characterise the spread in inferred parameter values and to assess the consistency of phenotype‐specific differences across independent optimisation runs. Rather than convergence to a single parameter set, robustness is evaluated in terms of the consistency of model fit quality and group‐level parameter differences across independent runs.

#### 2.2.6 One‐At‐a‐Time Parameter‐Substitution Experiments

We performed one‐at‐a‐time parameter substitution experiments to isolate the effect of individual parameters on model outputs during active standing that exhibited statistically significant differences between the two populations with and without a history of falls. As a baseline, we used the population‐average parameter set for participants without a history of falls, obtained from the best‐fitting run. For each parameter, we constructed a modified parameter vector by offsetting only that parameter in the baseline by the difference between the cohort means (fallers minus non‐fallers), leaving all other parameters unchanged. The resulting simulations were used to evaluate the local qualitative and quantitative influence of each parameter on the model outputs that showed statistically significant differences between populations with and without a history of falls during active standing.

## 3 Results

We present the results of our model fitting and parameter comparison analyses. The model fit performance is evaluated across multiple seeds, and the differences in key parameters between cohorts with and without a history of falls are examined.

### 3.1 Model Fit

**FIGURE 5:**
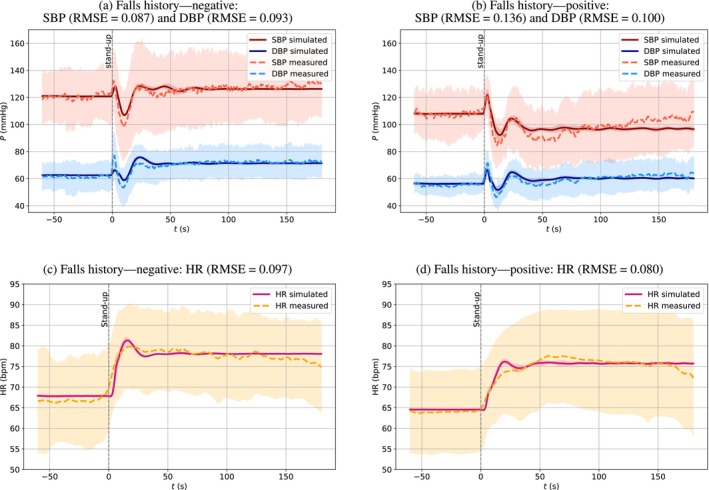
Measured and simulated (10 seeds) mean ± SD systolic blood pressure (SBP), diastolic blood pressure (DBP), and heart rate (HR) for falls history—negative (left, n=23, combined RMSE = 0.096) and falls history—positive (right, n=7, combined RMSE = 0.106). Standard deviations are shown in lighter shades of the corresponding traces; simulated SDs are barely visible due to their small magnitude. Definition: Positive = ≥ 1 fall in the previous year; negative = 0 falls.

Model‐fitted systolic and diastolic blood pressure and heart rate were compared with the corresponding measurements (Figure 5). The plots show the cohort means for participants with a history of falls (n=7) and those without such a history (n=23), alongside the mean of 10 independent data‐fitting runs. The combined RMSE between model and data was 0.096 for population without a history of falls and 0.106 for those with a history of falls (Appendix D Table A1), with individual‐output RMSEs ranging from 0.072 to 0.148 (Appendix D Table A2). The model results closely followed the averaged data curves and remained within the standard deviations of the measured signals. Discrepancies between model predictions and measured blood pressure or heart rate reflect limitations of the lumped parameter representation, inter‐individual variability, and unmodelled mechanisms (e.g., variable timing of skeletal muscle recruitment, heterogeneous vascular responses). These mismatches do not alter the general trends but should be considered when interpreting parameter estimates as system‐level indicators rather than uniquely identifiable physiological quantities.

The population with a history of falls exhibited lower blood pressure throughout the standing phase and a slower recovery towards baseline compared to the population without a history of falls, with incomplete return to baseline by the end of the measurement. Heart rate responses were also slower in those with a history of falls, showing a more gradual rise than in those without such a history. Considerable inter‐individual variability was present in both cohorts, as reflected by the wide standard deviations in the measured data.

We observed that the fitting procedure resulted in varying quality of fit across the entire temporal range of the response. Notably, the minimum systolic blood pressure at 10 s (Figure 5a,b) was, on average across seeds, approximately 6 mmHg higher than in the data. The diastolic minimum showed a similar but smaller offset of about 4 mmHg. In both populations, blood pressure exhibited an initial recovery followed by a second drop at 40–50 s, which was more pronounced in the population with a history of falls. The model–data mismatch was also greater during this second drop. The measured blood pressure from 50 s showed a slight but steady increase, again more evident in the population with a history of falls, whereas this delayed recovery was absent in the model, leading to a somewhat larger error. Finally, the model captured the initial peak in systolic and diastolic blood pressure immediately after stand‐up in the population with a history of falls. In contrast, in the population without a history of falls, the corresponding peak was slightly less pronounced in the model diastolic blood pressure compared to the measured data.

The modelled heart rate (Figure 5c,d) exhibited a recovery overshoot, with a small peak around 20 s, which was not observed in the averaged data. In addition, the resting heart rate differed slightly between cohorts: the heart rate was offset by approximately 0.5 bpm in the population with a history of falls and by 1 bpm in the population without a history of falls, compared to the model. The data also showed a small anticipatory rise in heart rate before standing (3 bpm from 9 s in the population without a history of falls and 1 bpm from 4 s in the population with a history of falls), which the model cannot capture. Furthermore, following the initial rise, the data showed a gradual, slow and steady decline in heart rate over time, which the model did not reproduce.

**FIGURE A2:**
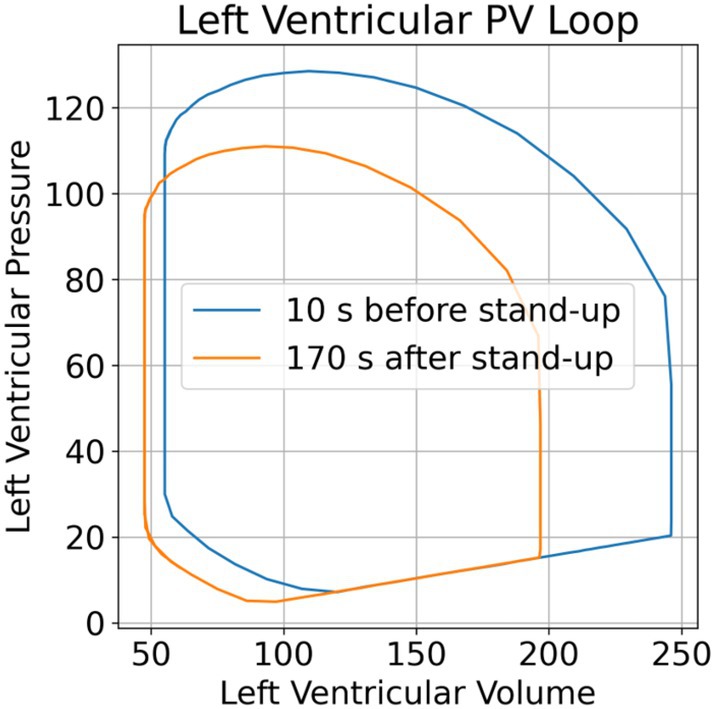
Left ventricular pressure–volume (PV) loop generated by the proposed cardiac model 10 s before and 170 s after stand‐up. Pressures shown in mmHg, volumes shown in mL.

**FIGURE A3:**
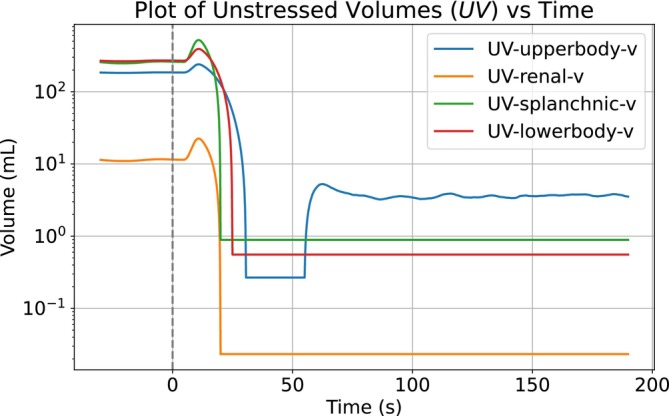
Time evolution of the unstressed volumes (UV) in selected vascular compartments (upper body, renal, splanchnic and lower body) during the sit‐to‐stand manoeuvre. The vertical dashed line indicates the onset of standing. A logarithmic scale is used on the vertical axis to visualise relative changes across compartments with different baseline volumes.

**FIGURE A4:**
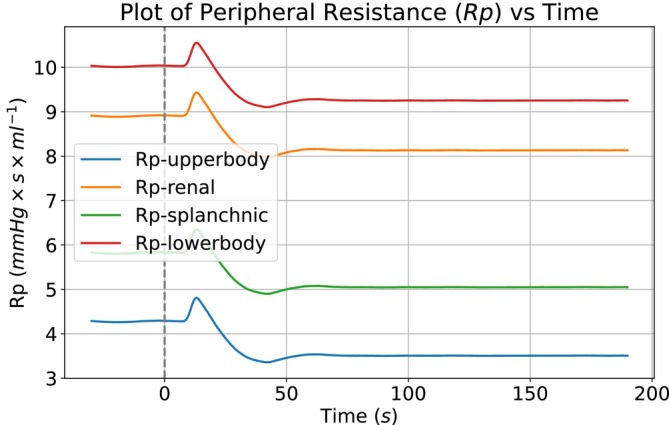
Time evolution of peripheral resistances ($R_{p}$) in the upper body, renal, splanchnic and lower body compartments during the sit‐to‐stand manoeuvre. The vertical dashed line indicates the onset of standing. The curves illustrate the rapid reflex‐mediated vasoconstriction and subsequent recovery following postural change.

**FIGURE A5:**
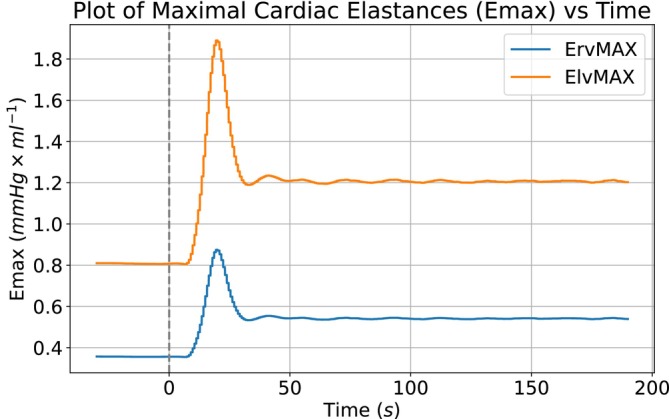
Time evolution of the maximal ventricular elastances ($E_{\mathrm{RV}}^{\max}$ and $E_{\mathrm{LV}}^{\max}$) during the sit‐to‐stand manoeuvre. The vertical dashed line indicates the onset of standing. The trajectories reflect reflex‐driven changes in cardiac contractility in response to the transient drop in arterial pressure after standing.

**FIGURE A6:**
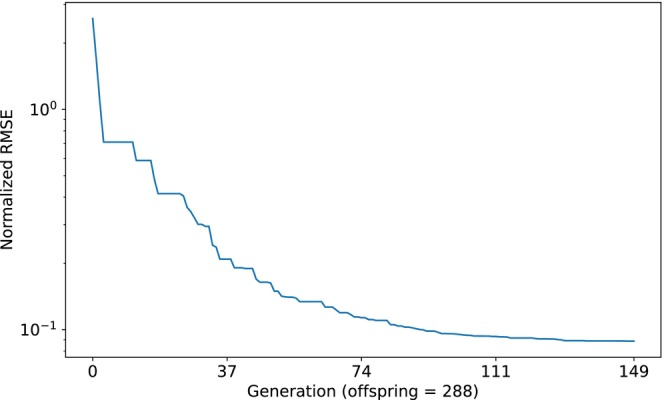
Normalized weighted RMSE (log‐scale) of the population average without a history of falls data fit (seed 4 in Table A1) plotted across generations. Final generation RMSE = 0.081.

Representative simulated dynamics of the fitted model, namely the left‐ventricular pressure‐volume loop and the reflex‐driven time courses of unstressed volume, peripheral resistance, and ventricular elastance during standing, are provided in Appendix C, Figures A2, A3, A4, A5.

### 3.2 Parameter Comparison

The interquartile ranges of the estimated parameters (Table 3) indicate moderate variability across optimisation runs. Approximately 35% of the parameters exhibited an interquartile range smaller than 30% of the mean value, about 29% fell between 30%–50%, and the remaining 35% exceeded 50%. Parameters that were comparatively well constrained included the sympathetic heart rate gain ($G_{\mathrm{HR}}^{\text{symp}}$), the baroreflex gain on left ventricular contractility ($G_{E_{\mathrm{LV}}^{\max}}^{\mathrm{BR}}$), the baroreflex shape parameter ($P_{\mathrm{BR}}^{\mathrm{SC}}$), the heart rate setpoint (${\mathrm{HR}}^{\text{setp}}$), as well as several mechanical parameters such as $E_{\mathrm{LV}}^{\min}$, the systemic resistance R, arterial elastance $E_{\mathrm{art}}$, total blood volume $V_{\mathrm{tot}}$, and the temporary external pressure $P^{\mathrm{ext},\text{temp}}$. In contrast, larger spreads were observed primarily among reflex‐related parameters, particularly several reflex gains, which are known to be weakly identifiable in closed‐loop cardiovascular models. Despite this variability in individual parameter estimates, the model errors of the fitted solutions remained within a narrow range (weighted RMSE 0.081–0.113, Table A1), indicating practical non‐uniqueness of the parameter estimates whereby different combinations of parameters can reproduce similar haemodynamic responses.

**TABLE 3:** Comparison of scaled parameter values by falls history.

Parameter	Unit	Falls history—positive	Falls history—negative	*p*‐value
Reflex				
Gain: $G_{\mathrm{HR}}^{\text{symp}}$	(s/mmHg)	0.00474 (0.00344, 0.00568)	0.00361 (0.00329, 0.00379)	0.162
Gain: $G_{\mathrm{HR}}^{\text{paras}}$	(s/mmHg)	0.00417 (0.00247, 0.00525)	0.00471 (0.00333, 0.00586)	0.427
Gain: $G_{E_{\mathrm{LV}}^{\max}}^{\mathrm{BR}}$	(1/mL)	0.0206 (0.0197, 0.0273)	0.0210 (0.0168, 0.0253)	0.734
Gain: $G_{E_{\mathrm{RV}}^{\max}}^{\mathrm{BR}}$	(1/mL)	0.0447 (0.0105, 0.0723)	0.0597 (0.0451, 0.0797)	0.345
Gain: $G_{R_{p}}^{\mathrm{BR}}$	(s/mL)	−0.119 (−0.151, −0.0801)	−0.0847 (−0.114, −0.0424)	0.212
Gain: $G_{R_{p}}^{\mathrm{CPR}}$	(s/mL)	1.07 (0.738, 1.33)	0.799 (0.689, 0.937)	0.140
Gain: $G_{V^{u}}^{\mathrm{BR}}$ ^a^	(mL/mmHg)	8.17 (3.97, 10.58)	6.70 (5.03, 8.17)	0.970
Gain: $G_{V^{u}}^{\mathrm{CPR}}$ ^a^	(mL/mmHg)	43.5 (11.37, 73.5)	29.4 (7.27, 31.11)	0.791
Delay: $\tau_{\mathrm{HR}}^{\text{paras}}$	(s)	1.74 (0.941, 2.62)	1.75 (0.896, 2.39)	0.678
Delay: $\tau_{\mathrm{HR}}^{\text{symp}}$	(s)	6.07 (5.06, 6.77)	5.83 (5.06, 6.72)	0.791
Delay: $\tau_{R_{p}}^{\mathrm{BR}}$	(s)	12.0 (10.8, 12.5)	13.4 (11.4, 15.3)	0.241
Delay: $\tau_{V^{u}}^{\mathrm{BR}}$	(s)	10.1 (6.86, 12.6)	11.5 (9.32, 14.8)	0.427
Delay: $\tau_{R_{p}}^{\mathrm{CPR}}$	(s)	18.0 (15.7, 19.9)	17.4 (16.4, 18.2)	0.571
Delay: $\tau_{V^{u}}^{\mathrm{CPR}}$	(s)	7.04 (5.41, 6.88)	7.28 (5.15, 7.23)	0.571
**Shape param.:** $P_{\mathrm{BR}}^{\mathrm{SC}}$	**(mmHg)**	**18.9 (13.2, 21.6)**	**36.7 (30.6, 41.7)**	**0.002**
Shape param.: $P_{\mathrm{CPR}}^{\mathrm{SC}}$	(mmHg)	11.1 (3.15, 16.9)	8.82 (2.62, 15.4)	0.910
Setpoint: $P_{\mathrm{car}}^{\text{setp}}$	(mmHg)	53.6 (42.7, 62.1)	46.4 (43.6, 48.3)	0.678
Setpoint: $P_{\mathrm{RA}}^{\text{setp}}$	(mmHg)	7.08 (4.26, 9.39)	7.49 (5.55, 9.53)	0.970
**Setpoint:** $HR^{\text{setp}}$	**(bpm)**	**77.3 (72.7, 80.2)**	**99.0 (92.5, 105)**	**0.000**
Other				
$E_{\mathrm{LV}}^{\max}$	(mmHg/mL)	2.10 (1.36, 2.27)	2.58 (1.64, 3.11)	0.241
$E_{\mathrm{LV}}^{\min}$	(mmHg/mL)	0.178 (0.174, 0.198)	0.181 (0.172, 0.192)	0.791
$E_{\mathrm{RV}}^{\max}$	(mmHg/mL)	1.45 (1.08, 1.77)	1.64 (1.47, 1.98)	0.385
$E_{\mathrm{RV}}^{\min}$	(mmHg/mL)	0.0751 (0.0638, 0.0862)	0.0731 (0.0523, 0.0906)	0.910
R ^a^	(mmHg ⋅ s/mL)	7.79 (7.14, 8.61)	7.19 (6.55, 8.05)	0.212
$R_{p}$ ^a^	(mmHg ⋅ s/mL)	5.05 (4.67, 6.00)	4.15 (2.99, 5.14)	0.307
$E_{\mathrm{art}}$ ^a^	(mmHg/mL)	8.10 (6.37, 9.48)	7.71 (6.71, 8.93)	0.521
$E_{\mathrm{ven}}$ ^a^	(mmHg/mL)	0.0378 (0.0272, 0.0433)	0.0473 (0.0322, 0.0631)	0.273
$V^{u}$ ^a^	(mL)	610 (445, 692)	477 (385, 538)	0.076
$V_{\mathrm{tot}}$	**(mL)**	**4.69e+03 (4.08e+03, 5.16e+03)**	**5.41e+03 (5.23e+03, 5.82e+03)**	0.045
$P^{\mathrm{ext},\text{temp}}$	**(mmHg)**	**23.0 (19.3, 28.9)**	**10.6 (7.42, 13.9)**	**0.004**
$P^{\mathrm{ext},\text{perm}}$	(mmHg)	10.0 (5.02, 12.1)	10.4 (2.14, 16.7)	0.910

*Note:* Values are reported as mean (25th, 75th percentile). p‐values are from the Mann–Whitney U test; bold values indicate statistically significant differences (p<0.05). Definition: Positive = ≥ 1 fall in the previous year; negative = 0 falls.

Abbreviations: ART, arterial; BR, baroreflex; CAR, carotid; CPR, cardiopulmonary reflex; HR, heart rate; LV, left ventricle; PARAS, parasympathetic; RA, right atrium; RV, right ventricle; symp, sympathetic; VEN, venous.

^a^ For these parameters the lower body variant is shown; however they have also been applied to every other systemic part of the model (arms, head, renal and splanchnic parts). See Table A3 in Appendix E for more details.

**FIGURE 6:**
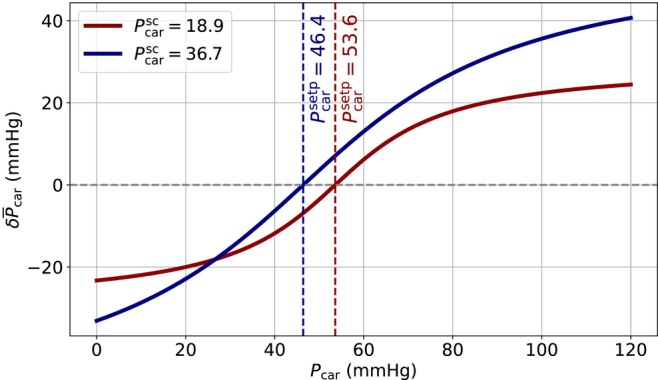
Scaled pressure difference $\delta {\bar{P}}_{\mathrm{car}}$ as a function of carotid pressure $P_{\mathrm{car}}$ for the population averages by falls history—positive ($P_{\mathrm{car}}^{\mathrm{sc}}=18.9$, $P_{\mathrm{car}}^{\text{setp}}=53.6$ [mmHg]) and falls history—negative ($P_{\mathrm{car}}^{\mathrm{sc}}=36.7$, $P_{\mathrm{car}}^{\text{setp}}=46.4$ [mmHg]). See Equation (8).

**FIGURE 7:**
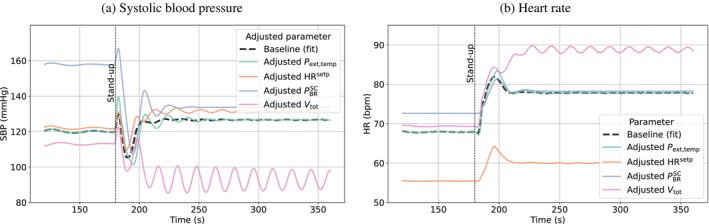
Comparison of seed 4 (falls history—negative) systolic blood pressure (SBP) and heart rate (HR) with single‐parameter–adjusted simulations moved toward falls history—positive cohort values by the differences reported in Table 3. Each panel shows the baseline model output (black, dashed) and the corresponding adjusted simulation (coloured, solid). Parameter values (baseline → adjusted): $P_{\mathrm{ext},\text{temp}}$ 15.6 → 33.9 (mmHg), ${\mathrm{HR}}^{\text{setp}}$ 91.6 → 71.5 (bpm), $P_{\mathrm{BR}}^{\mathrm{SC}}$ 41.8 → 21.5 (mmHg), $V_{\mathrm{tot}}$ 4040 → 3500 (mL).

Four parameters differ consistently between the two independently fitted cohort‐average datasets under repeated optimisation (Mann–Whitney U test, p<0.05): the baroreflex shape parameter $P_{\mathrm{BR}}^{\mathrm{SC}}$ (maps carotid pressure to a scaled pressure difference; see Equation (8)), the baroreflex heart‐rate setpoint ${\mathrm{HR}}^{\text{setp}}$ (baseline autonomic target; Equation (12)), the total blood volume $V_{\mathrm{tot}}$ (sets baseline filling/pressure; Equation (18)), and the transient external pressure applied by the muscles during standing $P_{\mathrm{ext},\text{temp}}$ (early mechanical compression; Equation (7)). These parameters were therefore selected for further model‐based interpretation. The resulting baroreflex shape functions for both cohorts are shown in Figure 6. The population with a history of falls exhibited a steeper transition and faster saturation at both lower and higher carotid pressures than those without such a history, whereas the latter appeared more linear across the pressure range. We performed one‐at‐a‐time parameter‐substitution experiments on these four parameters to explore their contributions to the differences between cohorts. Starting from a fitted simulation of the population without a history of falls (seed 4), we adjusted each parameter by the difference between the cohort means in Table 3 (Figure 7; diastolic blood pressure not shown, as the effects were similar to those for systolic blood pressure). This analysis is based on a single locally optimal parameter set and therefore represents a local, conditional exploration of model behaviour. When $P_{\mathrm{BR}}^{\mathrm{SC}}$ was increased, the pressure nadir deepened, the baseline shifted upward, and recovery was reduced; when $V_{\mathrm{tot}}$ was reduced, overall pressure decreased, the standing–rest difference increased, and heart rate during standing increased; when $P_{\mathrm{ext},\text{temp}}$ was reduced, the early blood‐pressure bump was attenuated; when ${\mathrm{HR}}^{\text{setp}}$ was altered, the heart‐rate level shifted with minimal direct effect on blood pressure. Overall, $P_{\mathrm{BR}}^{\mathrm{SC}}$ and $V_{\mathrm{tot}}$ exerted the largest effects on blood pressure, whereas $V_{\mathrm{tot}}$ and ${\mathrm{HR}}^{\text{setp}}$ primarily influenced heart rate.

## 4 Discussion

We aim to investigate physiologically interpretable model parameters that distinguish older adults with a history of falls from those without, to strengthen orthostatic hypotension assessment beyond static blood pressure cut‐offs. As a first step, our enhanced 23‐compartment closed‐loop cardiovascular model closely reproduced the population average blood pressure and heart rate responses to standing in both cohorts, providing a consistent phenomenological basis for subsequent cohort‐level interpretation. We then observed differences in model parameters related to reflexes and volume between those with a history of falls and those without. In one‐at‐a‐time substitution experiments, changing individual parameters from a set baseline shifted the simulated response from one phenotype to the other, indicating that specific regulatory mechanisms materially affect postural blood pressure dynamics. Our analysis shows that four model parameters carry consistent discriminatory information between cohorts under repeated optimization, despite non‐identifiability of the full parameter set, which influence baseline pressures and stand‐up responses mechanistically. These findings support the potential of model‐inferred dynamic markers to complement current diagnostics in older adults.

### 4.1 Fit Performance

Across ten independent seeds, the model achieved low normalised error in both cohorts, and simulated trajectories remained well within the empirical variability of the measurements, with markedly less dispersion than the between‐subject spread. The overall goodness‐of‐fit (normalised RMSE ≈10%) indicates that the model captures the dominant haemodynamic response to standing at the cohort level. The quality of fit varied over time. On average, the systolic and diastolic nadirs after standing were higher in the simulations than in the data, a clinically relevant discrepancy given the diagnostic role of the nadir in initial orthostatic hypotension [5]. The timing of the nadir matched the measurements and is consistent with prior descriptions of initial OH.

For heart rate, the simulations did not reproduce the small anticipatory increase observed prior to standing. This is consistent with the structure of the model, which represents closed‐loop cardiovascular regulation and does not include feed‐forward neural mechanisms (central command). Such anticipatory responses are not directly driven by cardiovascular feedback and cannot be identified from the available haemodynamic measurements alone.

In the later phase of the response, the model did not fully capture the secondary blood pressure dip around 40–50 s or the gradual recovery observed in both blood pressure and heart rate. These mismatches suggest that slower physiological mechanisms, such as delayed venous pooling, neuro‐humoral activation, and longer‐timescale autonomic adaptation, play an important role in shaping the late orthostatic response. As these processes are not included in the current model formulation, this represents a structural limitation when interpreting late‐phase dynamics [12].

Despite these phase‐specific discrepancies, the model reproduces the primary orthostatic dynamics and their timing with good accuracy. The parameter estimates should therefore be interpreted in relation to the fast cardiovascular control mechanisms explicitly represented in the model, rather than as descriptors of slower regulatory pathways that are not included.

### 4.2 Physiological Interpretation of Observed Parameter Differences

Four model parameters were significantly different between the populations (Mann–Whitney U test, p<0.05): (i) the baroreflex shape parameter $P_{\mathrm{BR}}^{\mathrm{SC}}$, (ii) the baroreflex heart rate setpoint ${\mathrm{HR}}^{\text{setp}}$, (iii) the total blood volume $V_{\mathrm{tot}}$, and (iv) the transient external pressure applied by muscles during standing, $P_{\mathrm{ext},\text{temp}}$. These differences were observed at the level of fitted model parameters and are interpreted here in a qualitative, model‐based manner, rather than as direct physiological estimates. To provide context, we first evaluate how these parameters relate to previously reported parameter sensitivities in lumped cardiovascular models, before discussing their physiological implications.

The total blood volume $V_{\mathrm{tot}}$ and the heart rate setpoint ${\mathrm{HR}}^{\text{setp}}$ correspond directly to parameters identified as highly sensitive in the local sensitivity analysis of Heldt [109], which dominate the regulation of mean arterial pressure, central venous pressure, stroke volume, and heart rate. The baroreflex shape parameter $P_{\mathrm{BR}}^{\mathrm{SC}}$ is not identical to parameters in Heldt's model, but Heldt also identified baroreflex‐related parameters as sensitive. In our model, $P_{\mathrm{BR}}^{\mathrm{SC}}$ modulates the strength and steepness of baroreflex responses and may therefore play a functionally analogous role in shaping time‐dependent blood pressure changes during standing. The transient external pressure applied by muscles during standing, $P_{\mathrm{ext},\text{temp}}$, is a model‐specific mechanism introduced in the present study. The observed differences in $P_{\mathrm{ext},\text{temp}}$ between cohorts hint that including this mechanism may capture aspects of orthostatic blood pressure regulation not represented in the original model, potentially reflecting relevant physiological processes. The physiological significance of these parameters is discussed in the following paragraphs.

The baroreflex shape parameter $P_{\mathrm{BR}}^{\mathrm{SC}}$ (Equation (8), Figure 6) governs the rate at which the baroreflex pressure sensors approach saturation. In normal blood pressure fluctuations, the reflex response (the slope of the function) does not differ noticeably between populations. However, under larger perturbations, the response saturates more rapidly in the population with a history of falls, as indicated by the smaller fitted value of $P_{\mathrm{BR}}^{\mathrm{SC}}$. Within the structure of the present model, this behaviour is consistent with reduced effective baroreflex sensitivity (BRS). Several studies have highlighted the link between BRS, OH, and falls in older adults. Reduced BRS was independently associated with greater systolic blood pressure declines upon standing, even after adjustment for baseline blood pressure, underscoring the contribution of impaired baroreflex function to OH [112]. Similarly, blunted BRS was shown to be a significant contributor to falls, pointing to inadequate cardiovascular responses during postural change as a key mechanism [113]. More recently, the rate of orthostatic blood pressure decline, closely tied to baroreflex dynamics, was found to be associated with both frailty and fall risk, suggesting that the temporal profile of blood pressure regulation may be more critical than the absolute nadir [114]. In line with these findings, age‐related reductions in baroreceptor sensitivity impair compensatory cardiovascular adjustments during orthostatic stress, thereby increasing susceptibility to OH and falls [115]. Ageing has been shown to decrease cardiovagal BRS, resulting in blunted reflex changes in heart rate in response to blood pressure variations, increased blood pressure variability, and impaired ability to respond to acute pressure changes [116]. Increasing age and OH (defined as meeting OH criteria during at least one supine‐to‐stand transition on continuous blood pressure monitoring) were associated with lower BRS. BRS decreased by approximately 1% per year of age and was about 30% lower in individuals with OH [117]. Comorbidities such as hypertension and diabetes may further reduce BRS, exacerbating the risk of OH and falls in older adults [118].

The baroreflex heart rate setpoint (${\mathrm{HR}}^{\text{setp}}$, Equation (12)) represents the intrinsic baseline around which heart rate is regulated in the model. ${\mathrm{HR}}^{\text{setp}}$ is a latent tuning parameter of the closed‐loop baroreflex controller and is not directly observable as a physiological resting heart rate. Physiologically, this setpoint can be influenced by the balance between sympathetic and parasympathetic control of the sinoatrial node. The absolute difference in heart rate between the populations is only 3 to 4 bpm in both the resting and standing phases. Upon standing, the heart rate of the cohort without a history of falls overshoots its final equilibrium before settling, while the heart rate of the cohort with a history of falls rises to the maximum without overshooting. In the population with a history of falls, ${\mathrm{HR}}^{\text{setp}}$ was fitted to a lower value of 77.3 bpm, 21.7 bpm lower than that of the opposite population. This apparent discrepancy between the small observed inter‐cohort differences in heart rate and the large fitted difference in ${\mathrm{HR}}^{\text{setp}}$ indicates that substantial compensation by other components of the reflex pathway (e.g., gains and delays) occurs within the model. Consequently, the absolute value of ${\mathrm{HR}}^{\text{setp}}$ is model‐dependent and should be interpreted only in a relative sense within the present modelling framework. Within the present model formulation, a lower fitted setpoint implies a reduced range for heart‐rate increase in response to orthostatic stress. This model‐based interpretation may be compatible with reduced parasympathetic withdrawal capacity, but alternative combinations of reflex gains and delays could lead to similar heart‐rate dynamics. This pattern is consistent with impaired BRS. Lower BRS has been shown to limit both the magnitude and timing of heart rate responses to postural change, thereby reducing cardiovascular compensation during standing [112, 113, 115, 116]. While the present study did not investigate heart rate variability, future analyses could examine whether separation of parasympathetic and sympathetic contributions supports this interpretation [119].

$V_{\mathrm{tot}}$, the total blood volume, was fitted to 4.69±1.08 L and 5.41±0.59 L in the populations with and without a history of falls, respectively. In the present model, a lower fitted total blood volume reflects reduced effective circulating volume. This reduction is consistent with hypovolaemia, a known contributor to orthostatic intolerance via diminished preload and impaired blood pressure compensation [120, 121]. Lower blood volume reduces venous return, preload, and stroke volume, thereby compromising cerebral perfusion during postural changes. Based on height and weight, Nadler's equation estimated $V_{\mathrm{tot}}$ at 4.91±0.50 L for the falls‐history group and 4.70±0.89 L for the non‐faller group. While these estimates do not exactly match the model‐derived values, they are of similar magnitude and show no statistically significant difference between groups. This comparison should be interpreted cautiously, as the model‐derived values represent effective volumes within the modelling framework rather than direct physiological measurements. Assessing diuretic use could clarify whether reduced intravascular volume contributes to impaired orthostatic adaptation in this cohort, and future studies may individualize total blood volume and distribution using patient height and weight.

The fitted temporary external pressure $P_{\mathrm{ext},\text{temp}}$ was 23.0±9.6 mmHg and 10.6±6.4 mmHg for the populations with and without a history of falls, respectively, matching the larger initial peak observed in the population with a history of falls. Within the present modelling framework, this parameter represents an effective description of the transient muscle‐pump contribution rather than a direct estimate of muscle‐generated pressure. This difference supports including the function in the model and aligns with the absence of such a peak in tilt‐table tests, where muscle activation is minimal [74, 122, 123]. Muscle‐generated pressure briefly increases preload, enhancing stroke volume and blood pressure, which is a compensatory mechanism in orthostatic intolerance [25, 26].

Using the same PROHEALTH sit‐to‐stand dataset that was used for model fitting in the present study, Wang et al. [124] reported that, in the PROHEALTH sit‐to‐stand manoeuvre [103, 104], the populations differed significantly in baseline sitting systolic blood pressure, diastolic blood pressure, and mean arterial pressure, as well as in the systolic and diastolic blood pressure nadirs. Interestingly, when they examined the supine‐to‐stand manoeuvre, similar trends were present, but these differences did not reach statistical significance. In comparison, our model‐based analysis suggests that a small subset of fitted parameters may differ consistently between cohorts across repeated optimisation runs, despite practical non‐uniqueness of the full parameter set. This should be interpreted as a possible mechanistic explanation rather than direct experimental evidence. These parameters, although not direct blood pressure measurements, influence baseline pressures and nadir responses mechanistically. As an illustrative, model‐based comparison with the findings of Wang et al., the results from one data‐fitting experiment (seed 4) are compared (Figure 7) to the same outputs while changing one parameter at a time according to the difference between the cohorts (Table 3). Both the baroreflex shape parameter and total blood volume affect the orthostatic blood‐pressure change: in the population without a history of falls, blood pressure returns to baseline, whereas in the adjusted simulations it remains 20–30 mmHg below baseline. Within this local parameter exploration, this effect can account for the baseline blood pressure differences reported by Wang et al. Moreover, the baroreflex shape parameter could explain the observed nadir differences, as the nadir magnitude approximately doubles when this parameter is adjusted by the difference between the two populations. Taken together, these results suggest that the reported cohort differences in baseline and nadir blood pressure are compatible with changes in baroreflex dynamics, heart‐rate regulation, circulating volume and muscle‐pump contributions as represented in the present model. Several factors may explain why Wang did not find statistically significant differences in the supine‐to‐stand manoeuvre, whereas our model‐based analysis did. (i) Model fitting can capture latent physiological differences that are not directly visible in raw pressure traces, making it more sensitive to subtle cohort distinctions. More precisely, the model integrates multiple dynamical features of the response into a constrained physiological representation. (ii) Our parameter estimates are informed by dynamic features of the response, such as the rate of change in heart rate or blood pressure and the presence of secondary dips, which Wang did not explicitly test for in her analysis. (iii) Finally, our analysis evaluates cohort‐level averages, which may uncover consistent differences that remain hidden in per‐subject comparisons due to inter‐individual variability.

### 4.3 Limitations

A limitation of this work is that several fitted parameters exhibit moderate interquartile ranges (approximately 20%–30% of the median values), while the fit error remains similar across different fitting trials with varying random seeds. We suspect that this is due to parameter redundancy, where one or more parameters can compensate for others to reproduce a similar model response. For example, the total unstressed volumes may be inversely correlated with the total blood volume in their effect on blood pressure and heart rate: a higher blood volume raises blood pressure, as does a lower unstressed volume. Parameter regression revealed some moderate correlations between parameter pairs (R^2^ > 0.75) in both population averages, but no consistently strong correlations (R^2^ > 0.95) between cohorts. The differing patterns between cohorts and the absence of very high R^2^ values suggest that any underlying redundancy may involve higher‐order relationships between multiple parameters rather than simple pairwise correlations. Such multi‐parameter interactions could allow different parameter sets to produce similar model outputs, consistent with practical non‐identifiability [125], although this has not been formally demonstrated. In this context, the reported cohort‐level differences should be interpreted as model‐based system‐level indicators under a fixed model structure, rather than as uniquely identifiable physiological parameter estimates.

The level of model detail adopted in the present study was guided by the physiological mechanisms described in Section 2.1.1. As a consequence, the model is not minimal, and alternative, more simplified model structures may also be able to capture similar dynamics. We therefore prioritise physiological interpretability and the ability to reproduce the dominant features of the active‐stand response, rather than minimising model complexity.

The present analysis is limited to the systemic cardiovascular contributors to orthostatic regulation. Falls in older adults are multifactorial, and cerebral autoregulation represents an additional mechanism not captured by the current model. Cerebral hypoperfusion may occur despite maintained systemic blood pressure in individuals with impaired autoregulatory capacity, and impaired cerebrovascular responses during orthostatic stress have been associated with fall risk independently of systemic blood pressure changes [126]. The framework is therefore intended to support mechanistic hypothesis generation and help disentangle the contribution of systemic cardiovascular regulatory mechanisms to orthostatic responses, rather than to provide a comprehensive or uniquely causal account of fall aetiology. Incorporation of cerebral haemodynamics and autoregulatory mechanisms into the modelling framework represents an important direction for future work.

Respiratory influences on cardiovascular regulation were not explicitly modelled; intrathoracic pressure was instead fixed at its mean value, omitting beat‐to‐beat effects of respiration on venous return and stroke volume [127] and on baroreflex modulation in closed‐loop cardiovascular analysis [128]. This simplification is supported by the attenuation of respiratory oscillations in population‐averaged signals and the lack of a consistent respiratory frequency peak in the haemodynamic waveforms. We do not expect the omission of respiratory modelling to contribute meaningfully to the observed fitting error (Section 4.1), as respiration primarily contributes to beat‐to‐beat variability.

### 4.4 Future Work

While the present study focused on cohort‐average data, the observed interquartile ranges indicate that some parameters exhibit notable spread between individuals. Future work will involve fitting the model to individual patients to enable personalised parameter identification, classification, and clustering analyses aimed at better predicting orthostatic hypotension and fall risk.

To improve parameter interpretability and reduce dimensionality, sensitivity analysis (SA) will be employed to identify insensitive parameters [129, 130] and detect parameters with similar sensitivities, which can then be removed or clustered. Principal component analysis of fitted parameters may also help reveal higher‐order correlations. Additionally, uncertainty quantification methods could be integrated to systematically assess confidence in parameter estimates and model predictions.

The data‐fitting procedure may be enhanced by combining the current approach with local data fitting or by using ISRES+ [131], and the cost function could be refined to place greater weight on accurately capturing the blood pressure nadir. Future work will also integrate the cerebral haemodynamics of arterial–arteriolar and venous cerebrovascular beds, along with regulation of cerebral arterioles [29]. Incorporating these components will allow the model to capture intracranial blood volume dynamics and cerebral autoregulatory responses more realistically. Parameters will then be estimated using near‐infrared spectroscopy (NIRS) measurements, enabling direct comparison between simulated and observed cerebral oxygenation changes, and improving the model's ability to link systemic cardiovascular control with cerebral hemodynamics for evaluating cerebral perfusion.

## 5 Conclusions

This study presents an extended cardiovascular model that successfully reproduces the key short‐term blood pressure and heart rate dynamics during active standing in population averages of subjects with and without a history of falls. By incorporating temporary external muscle pressure, the model captures the pronounced initial systolic and diastolic peaks observed. The model further reproduces the average population dynamics with good accuracy, although large inter‐individual variability remains to be explored. Discrepancies in the late‐phase blood pressure recovery indicate that additional slower physiological mechanisms are still missing from the current model structure. Four parameters were found to differ significantly between the two phenotypes: the baroreflex shape parameter, the baroreflex heart rate setpoint, the total blood volume, and the transient external pressure applied by muscles during standing.

The framework developed here provides a foundation for future investigation of individualised model parameters and for exploring potential mechanistic markers of orthostatic hypotension and fall risk. At present, the proposed approach is limited to population‐average fitting and is intended to support mechanistic interpretation rather than individual classification or clinical decision‐making. Future work will focus on resolving parameter non‐identifiability, improving data fit strategies, refining the cost function, and integrating late‐phase mechanisms to enable robust modelling of individual responses in larger clinical datasets.

## Author Contributions

Valeria Krzhizhanovskaya and Alfons G. Hoekstra designed the study. Lex M. van Loon developed the first version of the model. Joeri A. van de Sande improved this model, performed the experiments, and wrote the manuscript. Eveline P. van Poelgeest, Nathalie van der Velde, Marjolein Klop, and Jurgen A. H. R. Claassen provided clinical and physiological perspectives on the study. Marjolein Klop and Jurgen A. H. R. Claassen also built and preprocessed the dataset used in this work. All co‐authors reviewed the manuscript.

## Disclosure

The authors have nothing to report.

## Ethics Statement

The PROHEALTH dataset was approved by the Medical Ethics Committee (CMO Arnhem–Nijmegen). All participants provided written informed consent. The study was conducted in accordance with the Declaration of Helsinki. The present study used fully de‐identified data.

## Conflicts of Interest

The authors declare no conflicts of interest.

## Data Availability

The data that support the findings of this study are available from the Nijmegen Radboud University PROHEALTH study. Restrictions apply to the availability of these data, which were used under license for this study. Data are available at https://doi.org/10.34973/gtsd‐jk95 with permission from Nijmegen Radboud University.

## Appendix A Complete System of Equations

### Compartment Pressures

For each compartment n=1,…,23, the transmural pressure $P_{n}\left(t\right)$ is computed from its volume $V_{n}\left(t\right)$ and unstressed volume $V_{n}^{u}$. For intrathoracic compartments $n\in {ℐ}_{\text{intra}}$,

(A1)
$$P_{n}=E_{n}\left(V_{n}-V_{n}^{u}\right)+P_{\text{intra}}.$$

Three venous compartments are described by a nonlinear pressure–volume relation with external muscle pressures

(A2)
$$P_{11}=\frac{\tan\;\left(\frac{V_{11}-V_{11}^{u}}{2V_{\max,10}/\pi }\right)}{\pi \left(\frac{1}{E_{11}}\right)/\left(2V_{\max,10}\right)}+P_{\text{muscle},\mathrm{abd}},$$

(A3)
$$P_{13}=\frac{\tan\;\left(\frac{V_{13}-V_{13}^{u}}{2V_{\max,12}/\pi }\right)}{\pi \left(\frac{1}{E_{13}}\right)/\left(2V_{\max,12}\right)}+P_{\text{muscle},\mathrm{lb}},$$

(A4)
$$P_{14}=\frac{\tan\;\left(\frac{V_{14}-V_{14}^{u}}{2V_{\max,13}/\pi }\right)}{\pi \left(\frac{1}{E_{14}}\right)/\left(2V_{\max,13}\right)}+P_{\text{muscle},\mathrm{abd}}.$$

For the remaining extrathoracic linear compartments,

(A5)
$$P_{3}=E_{3}\left(V_{3}-V_{3}^{u}\right),$$

(A6)
$$P_{4}=E_{4}\left(V_{4}-V_{4}^{u}\right),$$

(A7)
$$P_{n}=E_{n}\left(V_{n}-V_{n}^{u}\right)+P_{\text{muscle},\mathrm{abd}},\;n\in \left\{7,8,9,10\right\},$$

(A8)
$$P_{12}=E_{12}\left(V_{12}-V_{12}^{u}\right)+P_{\text{muscle},\mathrm{lb}},$$

(A9)
$$P_{22}=E_{22}\left(V_{22}-V_{22}^{u}\right),$$

(A10)
$$P_{23}=E_{23}\left(V_{23}-V_{23}^{u}\right).$$

### Flow Equations

All flows are driven by pressure differences and hydrostatic pressure terms $P_{i}^{h}$ and are subject to valve‐like unidirectional constraints:

(A11)
$$F_{1}=\left\{\begin{array}{ll} \frac{P_{21}-P_{1}-P_{1}^{h}}{R_{1,1}}, & P_{21}-P_{1}-P_{1}^{h}>0,\\ 0, & \text{otherwise}, \end{array}\right.$$

(A12)
$$F_{2}=\max\;\left(0,\frac{P_{1}-P_{2}-P_{2}^{h}}{R_{1,2}}\right),$$

(A13)
$$F_{3}=\max\;\left(0,\frac{P_{2}-P_{3}-P_{3}^{h}}{R_{1,3}}\right),$$

(A14)
$$F_{4}=\max\;\left(0,\frac{P_{3}-P_{4}}{R_{1,4}}\right).$$

(A15)
$$F_{5}=\left\{\begin{array}{ll} \frac{P_{4}-P_{5}+P_{4}^{h}}{R_{1,5}}, & \left(P_{4}+P_{4}^{h}>P_{5}\right)\wedge \left(P_{5}>P_{\text{intra}}\right),\\ \frac{P_{4}-P_{\text{intra}}+P_{4}^{h}}{R_{1,5}}, & \left(P_{4}+P_{4}^{h}>P_{\text{intra}}\right)\wedge \left(P_{\text{intra}}>P_{5}\right),\\ 0, & \text{otherwise}. \end{array}\right.$$

(A16)
$$F_{6}=\left\{\begin{array}{cc} \frac{P_{5}-P_{16}+P_{5}^{h}}{R_{4,5}}, & P_{5}>P_{16},\\ \frac{P_{5}-P_{16}+P_{5}^{h}}{10R_{4,5}}, & \text{otherwise}, \end{array}\right.$$

(A17)
$$F_{7}=\max\left(0,\frac{P_{1}-P_{6}+P_{6}^{h}}{R_{1,6}}\right),$$

(A18)
$$F_{8}=\max\left(0,\frac{P_{6}-P_{7}+P_{7}^{h}}{R_{1,7}}\right),$$

(A19)
$$F_{9}=\frac{P_{7}-P_{8}+P_{8}^{h}}{R_{1,8}},$$

(A20)
$$F_{10}=\frac{P_{8}-P_{9}}{R_{1,9}},$$

(A21)
$$F_{11}=\frac{P_{9}-P_{14}-P_{9}^{h}}{R_{4,9}},$$

(A22)
$$F_{12}=\frac{P_{7}-P_{10}+P_{10}^{h}}{R_{1,10}},$$

(A23)
$$F_{13}=\frac{P_{10}-P_{11}}{R_{1,11}},$$

(A24)
$$F_{14}=\left\{\begin{array}{ll} \frac{P_{11}-P_{14}-P_{11}^{h}}{R_{4,11}}, & >0,\\ 0, & \text{otherwise}, \end{array}\right.$$

(A25)
$$F_{15}=\frac{P_{7}-P_{12}+P_{12}^{h}}{R_{1,12}},$$

(A26)
$$F_{16}=\frac{P_{12}-P_{13}}{R_{1,13}},$$

(A27)
$$F_{17}=\left\{\begin{array}{ll} \frac{P_{13}-P_{14}-P_{13}^{h}}{R_{4,13}}, & >0,\\ 0, & \text{otherwise}, \end{array}\right.$$

(A28)
$$F_{18}=\left\{\begin{array}{ll} \frac{P_{14}-P_{15}-P_{14}^{h}}{R_{1,15}}, & >0,\\ 0, & \text{otherwise}, \end{array}\right.$$

(A29)
$$F_{19}=\left\{\begin{array}{ll} \frac{P_{15}-P_{16}-P_{15}^{h}}{R_{4,15}}, & P_{15}>P_{16},\\ \frac{P_{15}-P_{16}-P_{15}^{h}}{10R_{4,15}}, & \text{otherwise}, \end{array}\right.$$

(A30)
$$F_{20}=\left\{\begin{array}{ll} \frac{P_{16}-P_{17}}{R_{1,17}}, & >0,\\ 0, & \text{otherwise}, \end{array}\right.$$

(A31)
$$F_{21}=\left\{\begin{array}{ll} \frac{P_{17}-P_{18}}{R_{4,17}}, & >0,\\ 0, & \text{otherwise}, \end{array}\right.$$

(A32)
$$F_{22}=\frac{P_{18}-P_{19}}{R_{1,19}},$$

(A33)
$$F_{23}=\left\{\begin{array}{ll} \frac{P_{19}-P_{20}}{R_{1,20}}, & >0,\\ \frac{P_{19}-P_{20}}{10R_{1,20}}, & \text{otherwise}, \end{array}\right.$$

(A34)
$$F_{24}=\left\{\begin{array}{ll} \frac{P_{20}-P_{21}}{R_{1,21}}, & >0,\\ 0, & \text{otherwise}. \end{array}\right.$$

(A35)
$$F_{25}=\max\left(0,\frac{P_{2}-P_{22}-P_{22}^{h}}{R_{1,22}}\right),$$

(A36)
$$F_{26}=\max\left(0,\frac{P_{22}-P_{23}}{R_{1,23}}\right),$$

(A37)
$$F_{27}=\left\{\begin{array}{ll} \frac{P_{23}-P_{5}+P_{23}^{h}}{R_{2,5}}, & \left(P_{23}+P_{23}^{h}>P_{5}\right)\wedge \left(P_{5}>P_{\text{intra}}\right),\\ \frac{P_{23}-P_{\text{intra}}+P_{23}^{h}}{R_{2,5}}, & \left(P_{23}+P_{23}^{h}>P_{\text{intra}}\right)\wedge \left(P_{\text{intra}}>P_{5}\right),\\ 0, & \text{otherwise}. \end{array}\right.$$

### Volume Balance

The compartment volumes satisfy

(A38)
$${\dot{V}}_{1}=F_{1}-F_{2}-F_{7},$$

(A39)
$${\dot{V}}_{2}=F_{2}-F_{3},$$

(A40)
$${\dot{V}}_{3}=F_{3}-F_{4},$$

(A41)
$${\dot{V}}_{4}=F_{4}-F_{5},$$

(A42)
$${\dot{V}}_{5}=F_{5}-F_{6},$$

(A43)
$${\dot{V}}_{6}=F_{7}-F_{8},$$

(A44)
$${\dot{V}}_{7}=F_{8}-F_{9}-F_{12}-F_{15},$$

(A45)
$${\dot{V}}_{8}=F_{9}-F_{10},$$

(A46)
$${\dot{V}}_{9}=F_{10}-F_{11},$$

(A47)
$${\dot{V}}_{10}=F_{12}-F_{13},$$

(A48)
$${\dot{V}}_{11}=F_{13}-F_{14},$$

(A49)
$${\dot{V}}_{12}=F_{15}-F_{16},$$

(A50)
$${\dot{V}}_{13}=F_{16}-F_{17},$$

(A51)
$${\dot{V}}_{14}=F_{11}+F_{14}+F_{17}-F_{18},$$

(A52)
$${\dot{V}}_{15}=F_{18}-F_{19},$$

(A53)
$${\dot{V}}_{16}=F_{6}+F_{19}-F_{20},$$

(A54)
$${\dot{V}}_{17}=F_{20}-F_{21},$$

(A55)
$${\dot{V}}_{18}=F_{21}-F_{22},$$

(A56)
$${\dot{V}}_{19}=F_{22}-F_{23},$$

(A57)
$${\dot{V}}_{20}=F_{23}-F_{24},$$

(A58)
$${\dot{V}}_{21}=F_{24}-F_{1},$$

(A59)
$${\dot{V}}_{22}=F_{25}-F_{26},$$

(A60)
$${\dot{V}}_{23}=F_{26}-F_{27}.$$

## Appendix B Data Preprocessing

Heart rate data derived from peak‐to‐peak Finapres beat‐to‐beat pressure measurements raw and cleaned data are shown in Figure A1.

## Appendix C Model Results Supplementary

This pressure–volume loop (Figure A2) illustrates the functional behaviour of the cardiac chambers and valves in the present lumped‐parameter model. Valve dynamics are represented by pressure‐gated unidirectional flow elements, such that flow occurs only when the upstream pressure exceeds the downstream pressure and reverse flow is not permitted. As a consequence, the ventricle undergoes isovolumetric contraction and relaxation phases when both inlet and outlet valves are closed, reproducing the essential functional role of the mitral and aortic valves without explicitly modelling leaflet mechanics.

This section shows representative model time courses of key reflex‐controlled parameters during a sit‐to‐stand transition for a single simulation (Figures A3, A4, A5). The figures illustrate how the reflex mechanisms dynamically redistribute unstressed blood volume, modulate peripheral resistances, and adjust cardiac contractility in response to orthostatic stress.

## Appendix D Data Fit Supplementary

The normalised, weighted RMSE of the population average without a history of falls, fit seed 4, is plotted across generations in Figure A6.

The RMSE of every data fitting procedure seed for subjects with and without a history of falls is shown in Tables A1 and A2.

**TABLE A1:** Final generation weighted RMSE values (the average of heart rate, systolic and diastolic blood pressure RMSE) for subjects with and without a history of falls across 10 seeds.

Seed	Falls history–negative	Falls history–positive
1	0.098	0.108
2	0.100	0.099
3	0.097	0.097
4	0.081	0.113
5	0.092	0.108
6	0.092	0.111
7	0.096	0.110
8	0.100	0.103
9	0.095	0.109
10	0.096	0.095
Mean ± SD	0.096 ± 0.006	0.106 ± 0.006

**TABLE A2:** Final generation RMSE values for heart rate (HR), systolic blood pressure (SBP), and diastolic blood pressure (DBP) for subjects with (F) and without (NF) a history of falls across 10 seeds.

Seed	HR (NF)	SBP (NF)	DBP (NF)	HR (F)	SBP (F)	DBP (F)
1	0.102	0.090	0.103	0.078	0.148	0.098
2	0.108	0.083	0.110	0.077	0.125	0.095
3	0.099	0.087	0.105	0.073	0.119	0.099
4	0.094	0.076	0.072	0.079	0.143	0.117
5	0.087	0.091	0.099	0.088	0.136	0.101
6	0.086	0.089	0.100	0.104	0.126	0.101
7	0.090	0.092	0.107	0.072	0.169	0.089
8	0.119	0.095	0.085	0.079	0.128	0.101
9	0.089	0.092	0.103	0.076	0.132	0.119
10	0.100	0.093	0.094	0.072	0.129	0.083
Mean ± SD	0.097 ± 0.010	0.087 ± 0.006	0.098 ± 0.010	0.080 ± 0.010	0.136 ± 0.014	0.100 ± 0.011

## Appendix E Data Fit Parameters

The data fitting parameters are described in Table A3.

**TABLE A3:** Data fitting parameters: Description and ranges used for model fitting.

Parameter	Description	Range
Reflex		
Gain: $G_{\mathrm{HR}}^{\text{symp}}$	Scales the sympathetic BR gain (Equation (13), Table A9)	(0.2–5.0)
Gain: $G_{\mathrm{HR}}^{\text{paras}}$	Scales the parasympathetic BR gain (Equation (13), Table A9)	(0.2–5.0)
Gain: $G_{E_{\mathrm{LV}}^{\max}}^{\mathrm{BR}}$	Scales the β‐BR $C_{\mathrm{LV}}$ gain (Equation (14), Table A9)	(0.2–5.0)
Gain: $G_{E_{\mathrm{RV}}^{\max}}^{\mathrm{BR}}$	Scales the β‐BR $C_{\mathrm{RV}}$ gain (Equation (15), Table A9)	(0.2–5.0)
Gain: $G_{R_{p}}^{\mathrm{BR}}$	Scales the α‐BR $R_{p}$ gain (Equation (16), Table A9)	(0.2–5.0)
Gain: $G_{R_{p}}^{\mathrm{CPR}}$	Scales the α‐CPR $R_{p}$ gain (Equation (16), Table A9)	(0.2–5.0)
Gain: $G_{V^{u}}^{\mathrm{BR}}$	Scales all 5 (n=3,5,10,12,14) α‐BR $V^{u}$ gains (Equation (17), Table A9)	(0.2–5.0)
Gain: $G_{V^{u}}^{\mathrm{CPR}}$	Scales all 5 (n=3,5,10,12,14) α‐CPR $V^{u}$ gains (Equation (17), Table A9)	(0.2–5.0)
Delay: $\tau_{\mathrm{HR}}^{\text{paras}}$	Translates the start, peak and end timings of the parasympathetic BR convolutional kernel equally (Equation (9), Table A8)	(0.0–3.0)
Delay: $\tau_{\mathrm{HR}}^{\text{symp}}$	Translates the start, peak and end timings of the sympathetic BR convolutional kernel equally (Equation (9), Table A8)	(−0.5–15.0)
Delay: $\tau_{R_{p}}^{\mathrm{BR}}$	Translates the start, peak and end timings of the α‐BR $R_{p}$ convolutional kernel equally (Equation (9), Table A8)	(−0.5–15.0)
Delay: $\tau_{V^{u}}^{\mathrm{BR}}$	Translates the start, peak and end timings of the α‐BR $V^{u}$ convolutional kernel equally (Equation (9), Table A8)	(−0.5–15.0)
Delay: $\tau_{R_{p}}^{\mathrm{CPR}}$	Translates the start, peak and end timings of the α‐CPR $R_{p}$ convolutional kernel equally (Equation (9), Table A8)	(−0.5–15.0)
Delay: $\tau_{V^{u}}^{\mathrm{CPR}}$	Translates the start, peak and end timings of the α‐CPR $V^{u}$ convolutional kernel equally (Equation (9), Table A8)	(−0.5–15.0)
Shape param.: $P_{\mathrm{BR}}^{\mathrm{SC}}$	Scales the baroreflex shape parameter (Equation (8), Table A9)	(0.2–5.0)
Shape param.: $P_{\mathrm{CPR}}^{\mathrm{SC}}$	Scales the cardiopulmonary reflex shape parameter (Equation (8), Table A9)	(0.2–5.0)
Setpoint: $P_{\mathrm{car}}^{\text{setp}}$	Scales the baroreflex carotid pressure setpoint (Equation (8), Table A9)	(0.5–2.0)
Setpoint: $P_{\mathrm{RA}}^{\text{setp}}$	Scales the cardiopulmonary reflex right atrium pressure setpoint (Equation (8), Table A9)	(0.5–2.0)
Setpoint: ${\mathrm{HR}}^{\text{setp}}$	Scales the baroreflex heart rate setpoint (Equation (13), Table A9)	(0.5–2.0)
Cardiac		
$E_{\mathrm{LV}}^{\max}$	Scales the maximal left ventricular elastance (Equation (4), Table A4)	(0.5–2.0)
$E_{\mathrm{LV}}^{\min}$	Scales the minimal left ventricular elastance (Equation (4), Table A4)	(0.5–2.0)
$E_{\mathrm{RV}}^{\max}$	Scales the maximal right ventricular elastance (Equation (4), Table A4)	(0.5–2.0)
$E_{\mathrm{RV}}^{\min}$	Scales the minimal right ventricular elastance (Equation (4), Table A4)	(0.5–2.0)
Global vascular scaling		
R	Scales all resistances (Equation (2), Table A6)	(0.5–2.0)
$R_{p}$	Scales all peripheral resistances (Equation (2), Table A6)	(0.5–2.0)
$E_{\mathrm{art}}$	Scales all arterial (n=1,2,4,7,8,9,11,13,19,23) elastances, cardiac elastances excluded (Equation (1), Table A4)	(0.5–2.0)
$E_{\mathrm{ven}}$	Scales all venous (n=3,5,6,10,12,14,15,16,20) elastances, cardiac elastances excluded (Equation (1), Table A4)	(0.5–2.0)
$V^{u}$	Scales all unstressed volumes (Equation (1), Table A7)	(0.5–2.0)
$V_{\mathrm{tot}}$	Scales the total blood volume (Equation (18), Table A10)	(0.8–1.25)
External pressure		
$P^{\mathrm{ext},\text{temp}}$	Scales the temporary external pressure exerted in the thorax, abdomen and lower body (Equation (7), Table A10)	(0.2–5.0)
$P^{\mathrm{ext},\text{perm}}$	Scales the permanently external pressure exerted in the lower body (Equation (7), Table A10)	(0.2–5.0)

*Note:* The ranges indicate the minimum and maximum values explored during the fitting procedure.

Abbreviations: art, arterial; BR, baroreflex; car, carotid; CPR, cardiopulmonary reflex; HR, heart rate; LV, left ventricle; paras, parasympathetic; RA, right atrium; RV, right ventricle; Symp, sympathetic; ven, venous.

## Appendix F Supporting Information

All parameters used prior to data fitting are given in the Tables A4, A5, A6, A7, A8, A9, A10 below.

**TABLE A4:** Compliances of vascular and cardiac compartments.

Compartment	Symbol	Min	Max	Unit
Upper thoracic artery	$C_{1}$	0.13	—	mL/mmHg
Upper limb arteries	$C_{2}$	0.10	—	mL/mmHg
Upper limb veins	$C_{3}$	3.50	—	mL/mmHg
Cerebral arteries	$C_{4}$	0.10	—	mL/mmHg
Cerebral veins	$C_{5}$	3.50	—	mL/mmHg
Superior vena cava	$C_{6}$	1.30	—	mL/mmHg
Thoracic aorta	$C_{7}$	0.10	—	mL/mmHg
Abdominal aorta	$C_{8}$	0.10	—	mL/mmHg
Renal arteries	$C_{9}$	0.21	—	mL/mmHg
Renal veins	$C_{10}$	5.00	—	mL/mmHg
Splanchnic arteries	$C_{11}$	0.20	—	mL/mmHg
Splanchnic veins	$C_{12}$	65.00	—	mL/mmHg
Lower body arteries	$C_{13}$	0.20	—	mL/mmHg
Lower body veins	$C_{14}$	22.00	—	mL/mmHg
Abdominal veins	$C_{15}$	1.30	—	mL/mmHg
Inferior vena cava	$C_{16}$	0.50	—	mL/mmHg
Right atrium	$C_{17}$	1.35	3.33	mL/mmHg
Right ventricle	$C_{18}$	0.77	19.29	mL/mmHg
Pulmonary arteries	$C_{19}$	3.40	—	mL/mmHg
Pulmonary veins	$C_{20}$	9.00	—	mL/mmHg
Left atrium	$C_{21}$	1.64	2.00	mL/mmHg
Left ventricle	$C_{22}$	0.40	9.69	mL/mmHg
Ascending aorta	$C_{23}$	0.28	—	mL/mmHg

*Note:* For cardiac chambers, both minimal and maximal compliances are shown.

**TABLE A5:** Lengths of vascular segments.

Compartment	Symbol	Value	Unit
Upper thoracic artery	$h_{1}$	4.5	cm
Upper limb arteries	$h_{2}$	30	cm
Upper limb veins	$h_{3}$	30	cm
Cerebral arteries	$h_{4}$	20	cm
Cerebral veins	$h_{5}$	20	cm
Superior vena cava	$h_{6}$	14.5	cm
Thoracic aorta	$h_{7}$	16	cm
Abdominal aorta	$h_{8}$	14.5	cm
Renal arteries	$h_{9}$	0	cm
Renal veins	$h_{10}$	0	cm
Splanchnic arteries	$h_{11}$	5	cm
Splanchnic veins	$h_{12}$	5	cm
Lower body arteries	$h_{13}$	106	cm
Lower body veins	$h_{14}$	106	cm
Abdominal veins	$h_{15}$	14.5	cm
Inferior vena cava	$h_{16}$	6	cm
Right atrium	$h_{17}$	0	cm
Right ventricle	$h_{18}$	0	cm
Pulmonary arteries	$h_{19}$	0	cm
Pulmonary veins	$h_{20}$	0	cm
Left atrium	$h_{21}$	0	cm
Left ventricle	$h_{22}$	0	cm
Ascending aorta	$h_{23}$	10	cm

**TABLE A6:** Resistances of vascular and cardiac compartments.

Compartment	Symbol	Inflow 1	Inflow 2	Inflow 3	Outflow 1	Outflow 2	Outflow 3
Upper thoracic artery	$R_{1}$	0.003	—	—	0.014	0.008	—
Upper limb arteries	$R_{2}$	0.014	—	—	2.45	—	—
Upper limb veins	$R_{3}$	2.45	—	—	0.11	—	—
Cerebral arteries	$R_{4}$	0.11	0.11	—	0.028	—	—
Cerebral veins	$R_{5}$	0.011	—	—	0.01	—	—
Superior vena cava	$R_{6}$	0.01	—	—	0.1	0.03	0.09
Thoracic aorta	$R_{7}$	0.1	—	—	4.1	—	—
Abdominal aorta	$R_{8}$	4.1	—	—	0.11	—	—
Renal arteries	$R_{9}$	0.03	—	—	3	—	—
Renal veins	$R_{10}$	3	—	—	0.07	—	—
Splanchnic arteries	$R_{11}$	0.09	—	—	4.5	—	—
Splanchnic veins	$R_{12}$	4.5	—	—	0.1	—	—
Lower body arteries	$R_{13}$	0.11	0.07	0.1	0.019	—	—
Lower body veins	$R_{14}$	0.019	—	—	0.008	—	—
Abdominal veins	$R_{15}$	0.008	0.028	—	0.006	—	—
Inferior vena cava	$R_{16}$	0.006	—	—	0.003	—	—
Right atrium	$R_{17}$	0.003	—	—	0.07	—	—
Right ventricle	$R_{18}$	0.07	—	—	0.006	—	—
Pulmonary arteries	$R_{19}$	0.006	—	—	0.01	—	—
Pulmonary veins	$R_{20}$	0.01	—	—	0.007	—	—
Left atrium	$R_{21}$	0.008	—	—	2.45	—	—
Left ventricle	$R_{22}$	2.45	—	—	0.11	—	—
Ascending aorta	$R_{23}$	0.007	—	—	0.003	0.011	—

*Note:* Inflow and outflow resistances are shown.

**TABLE A7:** Unstressed volumes of model compartments.

Compartment	Symbol	Value	Unit
Upper thoracic artery	$V_{1}^{u}$	5	mL
Upper limb arteries	$V_{2}^{u}$	16	mL
Upper limb veins	$V_{3}^{u}$	345	mL
Cerebral arteries	$V_{4}^{u}$	10	mL
Cerebral veins	$V_{5}^{u}$	300	mL
Superior vena cava	$V_{6}^{u}$	16	mL
Thoracic aorta	$V_{7}^{u}$	200	mL
Abdominal aorta	$V_{8}^{u}$	10	mL
Renal arteries	$V_{9}^{u}$	20	mL
Renal veins	$V_{10}^{u}$	30	mL
Splanchnic arteries	$V_{11}^{u}$	300	mL
Splanchnic veins	$V_{12}^{u}$	1146	mL
Lower body arteries	$V_{13}^{u}$	200	mL
Lower body veins	$V_{14}^{u}$	716	mL
Abdominal veins	$V_{15}^{u}$	79	mL
Inferior vena cava	$V_{16}^{u}$	33	mL
Right atrium	$V_{17}^{u}$	14	mL
Right ventricle	$V_{18}^{u}$	36	mL
Pulmonary arteries	$V_{19}^{u}$	160	mL
Pulmonary veins	$V_{20}^{u}$	430	mL
Left atrium	$V_{21}^{u}$	11	mL
Left ventricle	$V_{22}^{u}$	20	mL
Ascending aorta	$V_{23}^{u}$	21	mL

**TABLE A8:** Baroreflex (BR) and cardiopulmonary reflex (CPR) convolutional kernel timing parameters.

Parameter	Symbol	Start	Peak	End	Unit
Parasympathetic	$T_{p}^{\mathrm{BR}}$	0.59	0.70	1	s
β‐adrenergic	$T_{\beta }^{\mathrm{BR}}$	2.5	3.5	15	s
α‐adrenergic BR $R_{p}$	$T_{\alpha R_{p}}^{\mathrm{BR}}$	5.0	10	42	s
α‐adrenergic BR $V_{u}$	$T_{\alpha V_{u}}^{\mathrm{BR}}$	2.5	3.5	30	s
α‐adrenergic CPR $R_{p}$	$T_{\alpha R_{p}}^{\mathrm{CPR}}$	5.0	9.0	40	s
α‐adrenergic CPR $V_{u}$	$T_{\alpha V_{u}}^{\mathrm{CPR}}$	2.5	5.5	35	s

**TABLE A9:** Baroreflex (BR) and cardiopulmonary reflex (CPR) parameters.

Parameter	Symbol	Value	Unit
Gain: sympathetic heart rate	$G_{\mathrm{HR}}^{\text{symp}}$	0.012	s/mmHg
Gain: parasympathetic heart rate	$G_{\mathrm{HR}}^{\text{paras}}$	0.005	s/mmHg
Gain: left ventricle maximum elastance	$G_{E_{\mathrm{LV}}^{\max}}^{\mathrm{BR}}$	0.022	1/mL
Gain: right ventricle maximum elastance	$G_{E_{\mathrm{RV}}^{\max}}^{\mathrm{BR}}$	0.007	1/mL
Gain: Peripheral resistance (BR)	$G_{R_{p}}^{\mathrm{BR}}$	−0.05	s/mL
Gain: Peripheral resistance (CPR)	$G_{R_{p}}^{\mathrm{CPR}}$	0.5	s/mL
Gain: Upper limb veins unstressed volume (BR, e=3)	$G_{V^{u}}^{\mathrm{BR}}$	5.3	mL/mmHg
Gain: Upper limb veins unstressed volume (CPR, e=3)	$G_{V^{u}}^{\mathrm{CPR}}$	13.5	mL/mmHg
Gain: Cerebral veins unstressed volume (BR, e=5)	$G_{V^{u}}^{\mathrm{BR}}$	5.3	mL/mmHg
Gain: Cerebral veins unstressed volume (CPR, e=5)	$G_{V^{u}}^{\mathrm{CPR}}$	13.5	mL/mmHg
Gain: Renal veins unstressed volume (BR, e=10)	$G_{V^{u}}^{\mathrm{BR}}$	1.3	mL/mmHg
Gain: Renal veins unstressed volume (CPR, e=10)	$G_{V^{u}}^{\mathrm{CPR}}$	2.7	mL/mmHg
Gain: Splanchnic veins unstressed volume (BR, e=12)	$G_{V^{u}}^{\mathrm{BR}}$	13.3	mL/mmHg
Gain: Splanchnic veins unstressed volume (CPR, e=12)	$G_{V^{u}}^{\mathrm{CPR}}$	64	mL/mmHg
Gain: Lower body veins unstressed volume (BR, e=14)	$G_{V^{u}}^{\mathrm{BR}}$	6.7	mL/mmHg
Gain: Lower body veins unstressed volume (CPR, e=14)	$G_{V^{u}}^{\mathrm{CPR}}$	30	mL/mmHg
Shape param.: BR	$P_{\mathrm{BR}}^{\mathrm{SC}}$	18	mmHg
Shape param.: CPR	$P_{\mathrm{CPR}}^{\mathrm{SC}}$	5	mmHg
Setpoint: carotid artery	$P_{\mathrm{car}}^{\text{setp}}$	81	mmHg
Setpoint: right atrium	$P_{\mathrm{RA}}^{\text{setp}}$	6	mmHg
Setpoint: heart rate	${\mathrm{HR}}^{\text{setp}}$	70	bpm

*Note: e* is the effector compartment index.

**TABLE A10:** Other model parameters.

Parameter	Symbol	Value	Unit
Total simulation time	$T_{\mathrm{sim}}$	360	s
Onset of supine‐to‐stand manoeuvre	$t_{0}$	180	s
Supine‐to‐stand transition time	$T_{\text{stand}}$	6	s
Ever‐present thoracic pressure	$P_{\text{thrx}}^{\mathrm{ext},0}$	−3.0	mmHg
Temporary thoracic pressure	$P_{\text{thrx}}^{\mathrm{ext},\text{temp}}$	15	mmHg
Temporary abdominal pressure	$P_{\mathrm{abd}}^{\mathrm{ext},\text{temp}}$	30	mmHg
Temporary lower body pressure	$P_{l}^{\mathrm{ext},\text{temp}}$	30	mmHg
Permanent lower body pressure	$P_{l}^{\mathrm{ext},\text{perm}}$	5.0	mmHg
Gravity	g	9.81	m/s^2^
Blood density	ρ	1060	g/mL
Total blood volume	$V_{\mathrm{tot}}$	5250	mL
Maximum volume non‐linear compliance splanchnic veins	$V_{12}^{\max}$	1500	mL
Maximum volume non‐linear compliance lower body veins	$V_{14}^{\max}$	1000	mL
Maximum volume non‐linear compliance abdominal veins	$V_{15}^{\max}$	150	mL
Limit: peripheral resistance scaling	$\lim_{R}$	1000	—
Limit: venous unstressed volume scaling	$\lim_{\mathrm{UV}}$	1000	—
Heart period maximum	${\mathrm{HP}}^{\max}$	1.5	s
Heart period minimum	${\mathrm{HP}}^{\min}$	0.3	s
Reflex function moving average update rate	$S_{\text{gran}}$	16	Hz
Reflex function moving average time‐frame	$T_{\text{buffer}}$	0.25	s
Heart period section 1 parameter	$k_{\mathrm{AV}}$	0.12	s^1/2^
Heart period section 2 parameter	$k_{\mathrm{SA}}$	0.2	s^1/2^
Heart period section 3 parameter	$k_{\mathrm{SV}}$	0.3	s^1/2^
